# Biomarker correlates with response to NY-ESO-1 TCR T cells in patients with synovial sarcoma

**DOI:** 10.1038/s41467-022-32491-x

**Published:** 2022-09-08

**Authors:** Alexandra Gyurdieva, Stefan Zajic, Ya-Fang Chang, E. Andres Houseman, Shan Zhong, Jaegil Kim, Michael Nathenson, Thomas Faitg, Mary Woessner, David C. Turner, Aisha N. Hasan, John Glod, Rosandra N. Kaplan, Sandra P. D’Angelo, Dejka M. Araujo, Warren A. Chow, Mihaela Druta, George D. Demetri, Brian A. Van Tine, Stephan A. Grupp, Gregg D. Fine, Ioanna Eleftheriadou

**Affiliations:** 1 GlaxoSmithKline, Collegeville, PA USA; 2 National Cancer Institute, Bethesda, MD USA; 3 Memorial Sloan Kettering Cancer Center, New York, NY USA; 4 Weill Cornell Medical Center, New York, NY USA; 5 University of Texas/MD Anderson Cancer Center, Houston, TX USA; 6 City of Hope Comprehensive Cancer Center, Duarte, CA USA; 7 H. Lee Moffitt Cancer Center, Tampa, FL USA; 8 Dana-Farber Cancer Institute and Ludwig Center at Harvard, Boston, MA USA; 9 Washington University in St. Louis School of Medicine, St. Louis, MO USA; 10 Kelly Center for Cancer Immunotherapy, Division of Oncology, Children’s Hospital of Philadelphia and University of Pennsylvania, Philadelphia, PA USA

**Keywords:** Tumour biomarkers, Sarcoma, Predictive markers

## Abstract

Autologous T cells transduced to express a high affinity T-cell receptor specific to NY-ESO-1 (letetresgene autoleucel, lete-cel) show promise in the treatment of metastatic synovial sarcoma, with 50% overall response rate. The efficacy of lete-cel treatment in 45 synovial sarcoma patients (NCT01343043) has been previously reported, however, biomarkers predictive of response and resistance remain to be better defined. This post-hoc analysis identifies associations of response to lete-cel with lymphodepleting chemotherapy regimen (LDR), product attributes, cell expansion, cytokines, and tumor gene expression. Responders have higher IL-15 levels pre-infusion (*p* = 0.011) and receive a higher number of transduced effector memory (CD45RA- CCR7-) CD8 + cells per kg (*p* = 0.039). Post-infusion, responders have increased IFNγ, IL-6, and peak cell expansion (*p* < 0.01, *p* < 0.01, and *p* = 0.016, respectively). Analysis of tumor samples post-treatment illustrates lete-cel infiltration and a decrease in expression of macrophage genes, suggesting remodeling of the tumor microenvironment. Here we report potential predictive and pharmacodynamic markers of lete-cel response that may inform LDR, cell dose, and strategies to enhance anticancer efficacy.

## Abstract (web summary)

Biomarkers predictive of response to T cell therapy remain to be better defined. This study identifies potential predictive and pharmacodynamic markers of response to NY-ESO-1 T-cell therapy in a solid tumor that may inform lymphodepletion, cell dose, and strategies to enhance anticancer efficacy.

## Introduction

Chimeric antigen receptor (CAR) T-cell therapies have revolutionized the treatment for hematologic malignancies^1,2^, but have shown limited efficacy in solid tumors^3^. Clinical activity in solid tumors, including metastatic HPV-associated carcinomas, melanomas, synovial sarcomas (SS), and myxoid/round cell liposarcomas (MRCLS), has been observed with engineered T-cell receptor (TCR) T cells^4–9^. In contrast to CAR T cells which recognize cell surface antigens, TCR T cells are engineered with a TCR specific for peptides from intracellular cancer antigens presented by a human leukocyte antigen (HLA) molecule^1,10^. Letetresgene autoleucel (lete-cel; GSK3377794) consists of autologous CD4+ and CD8 + T-cells transduced with a high-affinity TCR recognizing the NY-ESO-1 (New York esophageal squamous cell carcinoma 1) antigen in complex with specific HLA-A*02 alleles^7^. NY-ESO-1-specific TCR T cells have shown robust clinical efficacy in SS. One clinical trial of retrovirally transduced NY-ESO-1 TCR T cells with interleukin (IL)−2 treatment (NCT00670748) demonstrated objective clinical responses in 61% of patients with SS^9^, and study NCT01343043 identified antitumor responses in up to 50% of advanced SS patients treated with lete-cel^7^.

SS is characterized by an SS18-SSX 1, 2, or 4 fusion protein formed by chromosomal translocations at t[X; 18] [p11;q11]. This, in the proper cell context, drives malignant transformation via epigenetic disturbances of the SWI/SNF complex^11,12^. The translocation also causes aberrant upregulation of NY-ESO-1, which is expressed in ~80% of SS tumors^13,14^. SS tumors have been characterized as immune deserts with exceptionally low immune cell infiltration compared with other soft tissue sarcoma (STS) subtypes^15–17^. Macrophages are the predominant infiltrates and are associated with poor prognosis in SS^16,18^. Poor immune cell infiltration, coupled with low tumor HLA expression and mutational burden^15,19,20^ likely render SS less susceptible to non-cellular immunotherapies, with rare responses observed with immune checkpoint inhibitors^21,22^. The challenges associated with immunotherapies in SS underscore the significance of the clinical responses reported with TCR T-cell therapies.

To further increase treatment efficacy, it is necessary to identify cell product characteristics and biomarkers predictive of response. Peak cell expansion and cytokine levels on day of and post-infusion have been linked to response or remission to CAR T cells in hematological malignancies^23,24^. However, few biomarker correlates have been identified for solid tumors with engineered cell therapies. We report findings from a post-hoc analysis of study NCT01343043, identifying specific attributes of lymphodepleting chemotherapy regimens (LDRs), cell dose, and cell product which impact in vivo cell expansion and correlate with clinical response. We also report preliminary trends of lete-cel treatment-induced decrease of macrophage gene expression within the tumor microenvironment (TME). The findings from this comprehensive dataset contribute towards the progressive understanding of mechanisms of response and resistance to TCR T-cell therapy in SS, a model for solid tumors, and provide considerations for further immunotherapy developments.

## Results

### Standard LDR creates favorable conditions for infused T cells and highlights benefits of higher cell dose

Patients with advanced or metastatic SS were enrolled to four cohorts designed to evaluate the impact of varying NY-ESO-1 expression levels and LDR on response (Table 1). Baseline patient characteristics were similar across cohorts (Table S1). The primary endpoint was overall response rate as assessed by investigators and was 50% in Cohort 1, as previously reported by D’Angelo, et al.^7^, 31% in Cohort 2, 20% in Cohort 3, and 27% in Cohort 4 (Table 1). Review by an independent committee was performed as a sensitivity analysis and the results agreed with a high concordance of 89%. Response rates were unchanged for Cohorts 1 and 3 and increased to 46% and 47% in cohorts 2 and 4 respectively (Table 1). The secondary efficacy endpoints included best overall response, duration of response (DoR), progression-free survival (PFS) and overall survival (OS). Best overall response and DoR were previously reported by D’Angelo, et al. for Cohort 1 and Ramachandran, et al. for all cohorts^7,8^. Here we report median DoR ranging from 8.6 to 32.1 weeks, median PFS ranging from 8.6 to 22.4 weeks and median OS ranging from 9.9 to 26.2 months across cohorts (Table 1). The safety profile of Cohort 1 was previously reported by D’Angelo, et al.^7^. Here we report on safety events across all cohorts. Observed safety events were expected and consistent with lymphodepletion and T-cell activation, including cytopenias and cytokine release syndrome (Table S2, Table S3). Eleven patients were eligible for a second infusion of lete-cel. Two patients responded post second infusion, while the remaining patients had stable disease with evidence of changes in tumor size (Table S4). This exploratory, post-hoc analysis focuses on correlates post first infusion and considers responders as patients whose tumors had a partial or complete response as assessed by investigators. Biomarker associations presented here that are significant with investigator-assessed response remained significant when analyzed with response as assessed by independent review committee.

**Table 1:** Summary of efficacy results across study cohorts

Parameter	Cohort 1 (*n* = 12)	Cohort 2 (*n* = 13)	Cohort 3 (*n* = 5)	Cohort 4 (*n* = 15)
NY-ESO-1 Expression (IHC Score)	HIGH 2+ or 3+ in ≥50% of tumor cells	LOW ≥1+ in ≥1% cells but not exceeding 2+ or 3+ in ≥50% cells	HIGH 2+ or 3+ in ≥50% of tumor cells	
Lymphodepletion regimen	STANDARD fludarabine (30 mg/m2 × 4D) and cyclophosphamide (1800mg/m2 × 2D)		CYCLOPHOSPHAMIDE ONLY (1800mg/m^2^ × 2D)	REDUCED fludarabine (30 mg/m2 × 3D) and cyclophosphamide (600 mg/m2 × 3D)
Median transduced cell dose (*10^9) (Range*10^9)^a^	3.60 (0.45–14.36)	2.42 (1.60–5.01)	3.02 (1.53–5.00)	2.40 (1.00–4.95)
Investigator Assessed^a^ (Primary Endpoint)				
Overall response rate^b^ n (%) (95% CI)	6 (50 %) (0.21–0.79)	4 (31%) (0.09–0.61)	1 (20%) (0.01–0.72)	4 (27%) (0.08–0.55)
Best Overall Response^c^ n(%)				
Complete Response	1 (8%)	0	0	0
Partial Response	5 (42%)	4 (31%)	1 (20%)	4 (27%)
Stable Disease	5 (42%)	7 (54%)	3 (60%)	10 (67%)
Progressive Disease	1 (8%)	1 (8%)	0	1 (7%)
Not evaluable	0	1 (8%)	1 (20%)	0
Median DoR (range), weeks	31.0 (13–72)	8.6 (8–13)	32.1 (32–32)	16.4 (14–94)
Median PFS^f^ (95% CI), weeks	15.4 (7.7–38.0)	13.1 (7.9–13.9)	8.6 (0.7–36.1)	22.4 (11.3–26.6)
Median OS (95% CI), months^d^	24.3 (8.5–48.8)	9.9 (3.9–19.6)	19.9 (8.8–NA)	26.2 (9.2–40.6)
Independent Review Committee^a,e^ Overall response rate^b^ n (%) (95% CI)	6 (50%) (0.21–0.79)	6 (46%) (0.19–0.75)	1 (20%) (0.01–0.72)	7 (47%) (0.21–0.73)

*CI* confidence interval, *DoR* duration of response, *NA* not available, *OS* overall survival, *PFS* progression-free survival, *IHC* immunohistochemistry, *NY-ESO-1*, New York esophageal squamous cell carcinoma 1.

^a^Data from modified intent-to-treat population: all patients that received lete-cel infusion.

^b^Proportion of patients with a confirmed CR or PR relative to total number of patients with 95% Clopper-Pearson confidence intervals.

^c^Recorded from the time of first T-cell infusion until disease progression.

^d^Data from Long-Term Follow-up Study, cut-off April 23, 2021.

^e^Review by an independent committee was performed as a sensitivity analysis for the primary endpoint which was based on investigator assessment.

^f^PFS was defined as the time from T-cell infusion to the earliest documentation of disease progression or death from any cause or surgical resection or start of prohibited medication.

To understand differences across cohorts, we evaluated the impact of NY-ESO-1 expression and LDR on response. Patients in Cohort 2 were eligible for enrollment based on a lower percentage and lower intensity of NY-ESO-1 tumor staining (Table 1). We found most patients enrolled in Cohort 2 had ≥30% of tumor cells positive for NY-ESO-1 (Fig. S2a) and the median NY-ESO-1 expression across all cohorts was 80%. This high expression of NY-ESO-1 in SS is consistent with published reports^13^ and showed no significant impact on response (Fig. S2a).

**Fig. 1:**
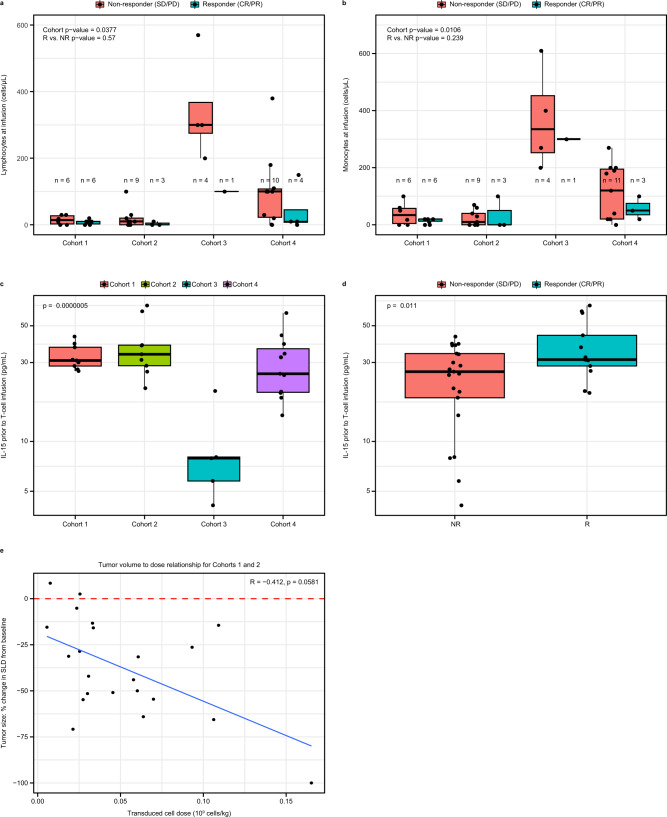
Standard LDR of fludarabine and cyclophosphamide forms supportive environment for T cells and highlights impact of cell dose. **a**, **b** Endogenous lymphocyte (**a**) and monocyte (**b**) cell counts across cohorts on day of infusion (*n* = 43 biologically independent samples). All responders except two in Cohorts 3 and 4 had <10 lymphocytes/μL prior to T-cell infusion. c,d, IL-15 levels prior to T-cell infusion across cohorts (**c**) and association with response (**d**) (*n* = 34 biologically independent samples). Values are log-transformed for consistency with other cytokine analyses. ANOVA and *t*-test were performed in **c** and **d** to be consistent with remaining cytokine analyses; Kruskal–Wallis *p*-value = 0.005 for (**c**) and Wilcoxon *p*-value = 0.050 for **d**. **e** Impact of transduced cell dose/kg on reduction in tumor size (*n* = 22 biologically independent samples) for patients in cohorts 1 and 2. Box plots depict median as horizontal line within box, with box bounds as the first and third quartiles. Dots represent individual data points. Lower whisker is the minimum value of the data within 1.5 times the interquartile range below the 25th percentile. Upper whisker is the maximum value of the data within 1.5 times the interquartile range above the 75th percentile. Two-sided *p*-values were calculated via bootstrapped median regression (10,000 bootstraps) to adjust for cohort or responder status (**a**, **b**), ANOVA (**c**), *t*-test (**d**), and standard test for Spearman correlation (**e**). CR complete response, IL interleukin, LDR lymphodepleting chemotherapy regimen, PD progressive disease, PR partial response, SD stable disease, SLD sum of longest diameter.

The proposed role of LDRs is to reduce elements of the endogenous immune system that compete with infused T cells for supportive cytokines^25,26^. Depletion of endogenous lymphocytes and monocytes prior to infusion varied significantly by cohort, with more complete depletion observed with standard LDR (Fig. 1a, b). IL-15 and IL-7 are cytokines which support T-cell proliferation and survival^26^ and studies have shown that these cytokines are increased post LDR, especially by fludarabine^8,24,27,28^. Consistent with this, we observed that patients that received LDRs containing fludarabine had higher IL-15 levels prior to T-cell infusion (Fig. 1c). The role of fludarabine was further demonstrated by data from two patients who received cyclophosphamide-only LDR for the first infusion and fludarabine-containing LDR for the second infusion. These patients had 2- and 7-fold increase in IL-15 respectively (Fig. S2b). Furthermore, across the cohorts responding patients had higher levels of IL-15 prior to T-cell infusion (Fig. 1d). There was no association of IL-7 pre-infusion with response (Fig. S2c). Use of standard LDR appeared to create favorable conditions for T-cell proliferation.

With a proliferation-permissive environment established by a standard LDR, we evaluated the impact of transduced cell dose (hereafter cell dose) on tumor reduction in patients from Cohorts 1 and 2. As shown in Fig. 1e, there was an inverse linear relationship of cell dose normalized by body weight with tumor volume reduction, which trended towards significance (*p* = 0.058). There was a trend of responders receiving a higher cell dose per kg (cell dose/kg) compared to non-responders (median 0.059 × 10^9/kg and 0.027 *10^9/kg respectively; *p* = 0.088) (Fig. S2d). This analysis controlled for any effects of LDR on tumor size, since patients included received the same LDR. These data demonstrate that a higher cell dose per kg in combination with standard LDR may offer opportunities to maximize antitumor efficacy.

### Standard LDR and higher weight-normalized cell dose are associated with higher peak cell expansion which is a marker of response

**Fig. 2:**
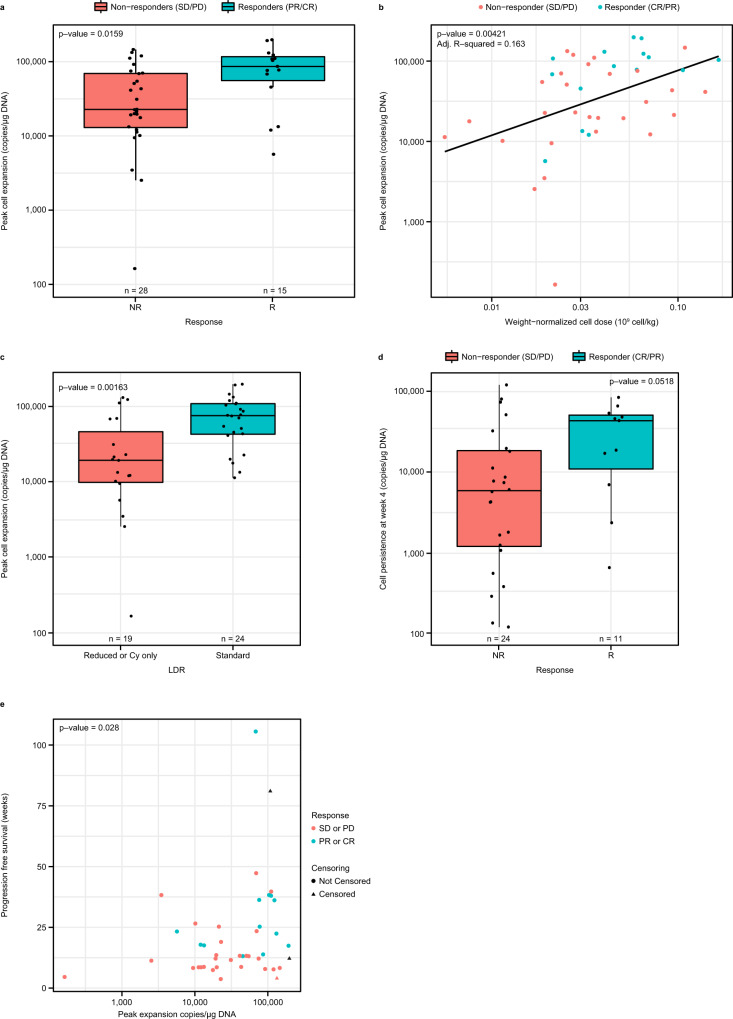
Lete-cel expansion post-infusion associated with response, cell dose/kg, and LDR. **a** Association of peak cell expansion (Cmax) with response (*n* = 43 biologically independent samples). b,c, Relationship of Cmax with weight-normalized cell dose with line of best fit in blue (**b**) and LDR (**c**) (*n* = 43 biologically independent samples). **d** Persistence of lete-cel at Week 4 post-infusion in all 4 cohorts, stratified by response (*n* = 35 biologically independent samples). **e** Relationship between Cmax and PFS (*n* = 43 biologically independent samples). PFS was defined as the time from T-cell infusion to the earliest documentation of disease progression or death from any cause or surgical resection or start of prohibited medication. Box plots depict median as horizontal line within box, with box bounds as the first and third quartiles. Dots represent individual data points. Lower whisker is the minimum value of the data within 1.5 times the interquartile range below the 25th percentile. Upper whisker is the maximum value of the data within 1.5 times the interquartile range above the 75th percentile. Two-sided *p*-values were calculated via Wilcoxon test (**a**, **c**, **d**) or linear (**b**) regression, and Cox proportional hazards model (**e**). CR complete response, Cy cyclophosphamide, LDR lymphodepleting chemotherapy regimen, PD progressive disease, PFS progression-free survival PR partial response, SD stable disease.

Following evaluation of pre-infusion correlates, we analyzed the expansion of lete-cel post-infusion and found responders across cohorts had higher peak cell expansion (Cmax) (*p* = 0.016, Fig. 2a), consistent with what was previously reported for Cohort 1^7^. Higher Cmax was associated with standard LDR (*p* = 0.00163) and higher weight-normalized cell dose (*p* = 0.00421) (Fig. 2b, c). The association of absolute dose with Cmax was weaker though still present (Fig. S3a). There was a trend towards higher persistence levels at Week 4 in responders (*p* = 0.0518) (Fig. 2d). Area under the cell expansion curve from day 0 to day 28 was closely correlated with Cmax and had similar association with response (Fig. S3b, c). There were no associations between baseline tumor burden and NY-ESO-1 expression with Cmax or response (Fig. S3d–f). In addition to response, higher Cmax was associated with longer progression-free survival (PFS) (*p* = 0.028) (Fig. 2e). Cell dose was not associated with PFS (Fig. S3g). These data further support the use of a standard LDR and the benefits of providing a higher cell dose/kg, when possible, to enable T cell expansion post infusion in synovial sarcoma.

### Responders received lete-cel product enriched with activated, effector memory CD8 cells and containing comparable total CD8 to CD4 cell ratio

Lete-cel contains a mixture of transduced and non-transduced T cells. In this study, the median transduction efficiency was 35% (range 14–65%). To characterize the transduced cells, we used an NY-ESO-1 major histocompatibility complex (MHC) class 1 pentamer reagent. This pentamer is primarily used to stain CD8 cells due to co-receptor stabilization^29^, therefore our analysis focused on CD8 + Pentamer+ cells.

**Fig. 3:**
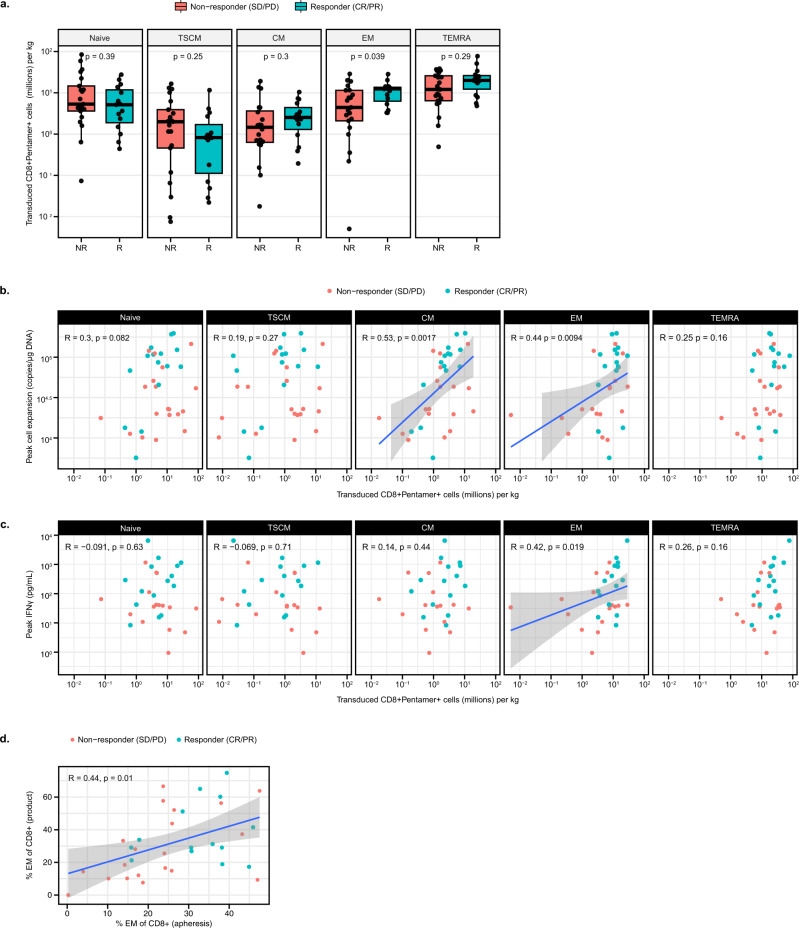
T-cell product enriched with activated, effector memory CD8 cells is associated with response. **a** Number of CD8 + Pentamer+ cells/kg infused per memory phenotype in non-responders vs responders (*n* = 36 biologically independent samples). **b**, **c** Association of number of infused cells/kg per memory phenotype with peak cell expansion (**b**) and peak IFNγ post-infusion (**c**) (*n* = 34 and 31 biologically independent samples for parts **b** and **c** respectively). Associations with EM cells are statistically significant with outlier at lowest infused cell counts removed—EM with Cmax (*R* = 0.42, *p* = 0.016) and EM with IFNγ (*R* = 0.41, *p* = 0.027). **d** Association of EM phenotype between apheresis and product (*n* = 34 biologically independent samples). Box plots depict median as horizontal line within box, with box bounds as the first and third quartiles. Dots represent individual data points. Lower whisker is the minimum value of the data within 1.5 times the interquartile range below the 25th percentile. Upper whisker is the maximum value of the data within 1.5 times the interquartile range above the 75th percentile. Nominal two-sided *p*-values based on the Wilcoxon rank sum test or log-rank test for PFS are presented and correlations are based on Spearman method. Line of best fit shown in blue for significant associations and gray area represents 95% confidence interval around the regression line. CM central memory (CD45RA-CCR7+), CR complete response, EM effector memory (CD45RA-CCR7-), Naïve (CD45RA + CCR7+), PFS progression-free survival, PD progressive disease, PR partial response, SD stable disease, TEMRA T effector memory RA (CD45RA + CCR7−), TSCM T stem cell memory (CD45RA + CCR7 + CD45RO-CD95 + CD127+).

On average, responders had 28% CD8 + Pentamer+ cells with an effector memory (EM) phenotype (CD45RA- CCR7-) (Fig. S4a, b). Since cell dose/kg has been shown to have an impact on response (Fig. 1e), we consider it important to analyze the absolute number of cells per phenotype infused. Notably, responders received a higher number of CD8 + Pentamer+ EM cells/kg, with a median of 12 million/kg compared to 4 million/kg for non-responders (Fig. 3a). No significant difference in other memory phenotype cells, such as T stem cell memory (TSCM) or central memory (CM), received by responders and non-responders, was observed in this study. A similar trend was observed when further analyzed as a logistic regression (Fig. S4c). Additionally, the number of CD8 + EM and CM cells infused were associated with Cmax (*p* = 0.0094 and *p* = 0.0017, respectively) (Fig. 3b). As T cells differentiate from naïve to terminal effector cells, their specialized function drives production of certain cytokines. We analyzed the relationship between infused memory phenotypes and levels of interferon (IFN)γ, a key cytokine for promoting host-immune recruitment to tumors^30^. Figure 3c shows an association between the number of CD8 + EM cells infused and peak IFNγ post-infusion (*p* = 0.019). This is an interesting finding, suggesting a link between the product phenotype and the functionality of these cells post-infusion.

To better understand factors that affect product attributes, the impact of the starting apheresis material was evaluated. There was a significant association between percent CD8 + EM in apheresis material and product (*p* = 0.01), with the majority of responders starting with >30% CD8 + EM (Fig. 3d, Fig. S4d). This highlights the possibility of the apheresis material to influence product characteristics and potentially identify patients who are more likely to respond. Further analysis is needed to identify patient characteristics that may influence the starting proportion of EM cells.

**Fig. 4:**
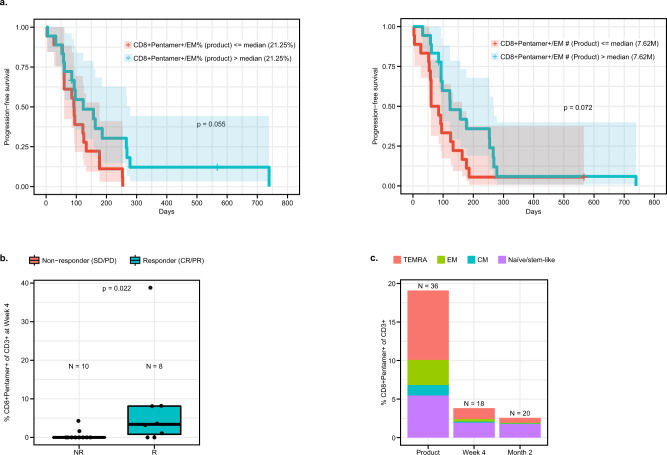
Characterization of transduced T cells post infusion. **a** Association of % and number of CD 8+  Pentamer+EM cells/kg with PFS.PFS was defined as the time from T-cell infusion to the earliest documentation of disease progression or death from any cause or surgical resection or start of prohibited medication (*n* = 36 biologically independent samples). Blue and red shaded areas represent 95% confidence interval for the survival curve. **b** Levels of CD8 + Pentamer+ cells at Week 4. **c** Levels and composition of memory phenotypes of CD8 + Pentamer+ cells post-infusion. Product analyses consist of 36 patients. Number of patients for post-infusion analyses are listed on figures. Box plots depict median as horizontal line within box, with box bounds as the first and third quartiles. Dots represent individual data points. Lower whisker is the minimum value of the data within 1.5 times the interquartile range below the 25th percentile. Upper whisker is the maximum value of the data within 1.5 times the interquartile range above the 75th percentile. Nominal two-sided *p*-values based on the Wilcoxon rank sum test or log-rank test for PFS are presented and correlations are based on Spearman method. CM central memory (CD45RA-CCR7+), CR complete response, EM effector memory (CD45RA-CCR7-), Naïve (CD45RA + CCR7+), PFS progression-free survival, PD progressive disease, PR partial response, SD stable disease, TEMRA T effector memory RA (CD45RA + CCR7-), TSCM T stem cell memory (CD45RA + CCR7 + CD45RO-CD95 + CD127+).

Together these findings demonstrate that CD8 + EM cells are important for lete-cel response in SS. When the impact of CD8 + EM cells on PFS was analyzed, there was only a trend toward increased PFS with higher percentage and number of infused cells/kg (*p* = 0.055 and *p* = 0.072, respectively) (Fig. 4a). This points to additional factors or cell populations which may be needed to achieve prolonged response. At Week 4, responders had higher levels of CD8 + Pentamer+ cells (Fig. 4b), consistent with DNA-based persistence results (Fig. 2d, S5a). Of these remaining cells, ~50% were characterized by a T naïve/stem-like phenotype (CD45RA + CCR7+) (Fig. 4c), suggesting this cell type may be beneficial for long-term persistence.

Further analysis of the product revealed responders received cells characterized by an activated phenotype and comparable ratio of total CD8+ to CD4+. Cells expressed CD40 ligand (CD40L) which was associated with an EM phenotype (Fig. S5b, c). There was no association of PD-1, LAG-3, CTLA-4, or TIM-3 expression levels in the apheresis and product with response (Fig. S5d, e); differential expression of TIM-3 between apheresis and product suggests upregulation as a result of activation during the manufacturing process. The comparable ratio of total CD8+ to CD4 + cells in responders suggests CD4 + cells may be important to support CD8 function (Fig. S5f).

Collectively, these data illustrate that a TCR T-cell product enriched with activated, EM CD8 cells is associated with response.

### Proinflammatory cytokines identified as pharmacodynamic markers associated with response

**Fig. 5:**
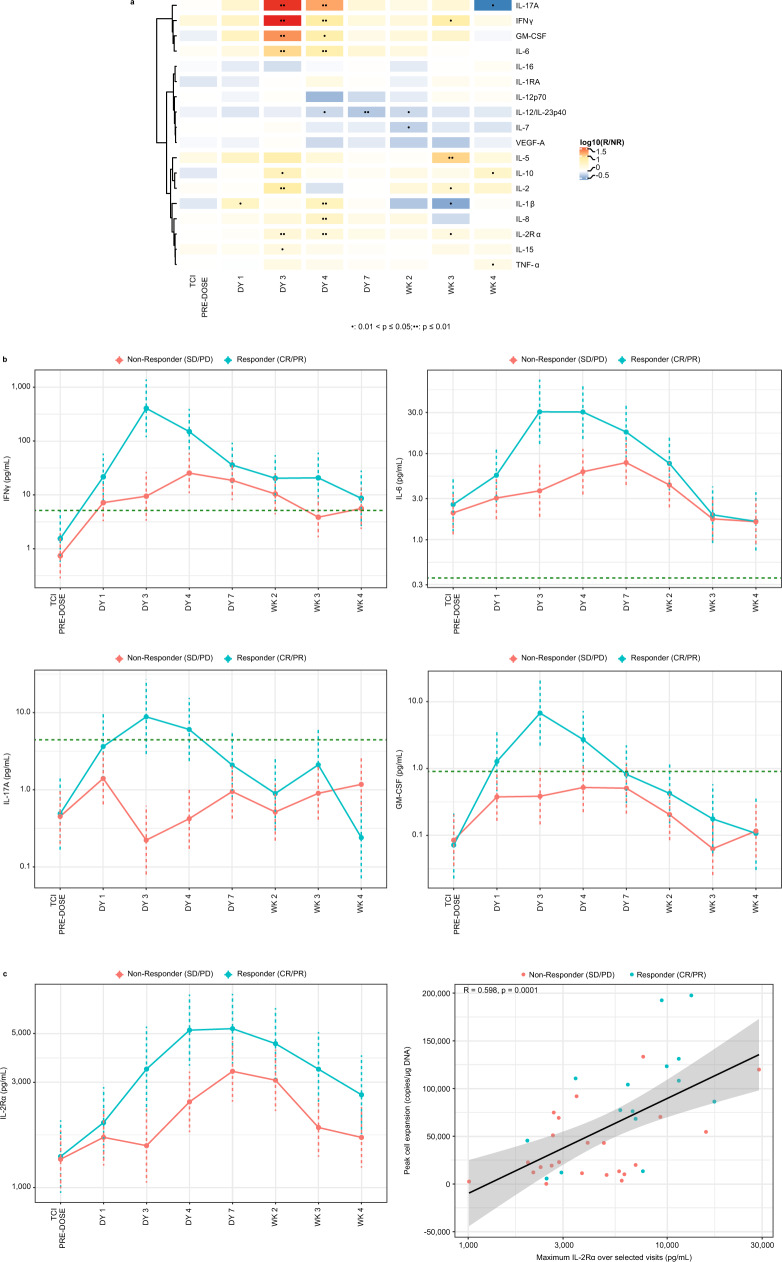
Increase in proinflammatory cytokines in responders post lete-cel infusion and correlation to peak cell expansion. **a** Heatmap of Responder vs Nonresponder ratios in cytokine levels over time obtained from linear mixed effects (LME) model. T-cell infusion (TCI) occurred on Day 0. Nominal *p*-values from linear effects models were calculated via Wald test and t-distribution and are shown as one dot for 0.01 < *p* ≤ 0.05 and two dots for *p* ≤ 0.01. **b** Time courses of IFNγ, IL-6, IL-17A, and GM-CSF, cytokines differentially upregulated by responders. Geometric means and 95% confidence intervals from LME model (accounting for left-censoring where appropriate) are plotted. Dashed, horizontal lines represented lower limit of quantification. **c** Time course of soluble IL-2Rα levels showing increase in responders and correlation to peak cell expansion. Line of best fit shown in black and gray area represents 95% confidence bands, both from standard least-squares regression. Sample size varied across timepoints and cytokines, with an overall median of 32 patients (range, 15–38 depending on cytokine/timepoint). For **c**, Spearman correlation and corresponding two-sided *p*-value are presented. DY day, GM geometric mean, GM-CSF granulocyte-macrophage colony-stimulating factor, IFN interferon, IL interleukin, WK week.

Post lete-cel infusion, several proinflammatory cytokines were significantly increased in responders as compared with non-responders at Day 3 and 4 (Fig. 5a, b). IFNγ, IL-6, and soluble IL-2 receptor alpha (IL-2Rα) were the most robust pharmacodynamic (PD) markers, while granulocyte-macrophage colony-stimulating factor (GM-CSF) and IL-17A were often present at levels below limit of quantification. Expression of these cytokines demonstrates that T cells become activated within a few days post-infusion and this occurs earlier in responders. The increased IFNγ in responders is intriguing as it highlights the impact of EM cells in the product which are associated with IFNγ production, and subsequently with response (Fig. 3a, c). The peak expression of soluble IL-2Rα showed the strongest correlation to Cmax, indicating a PK/PD relationship (Fig. 5c). Strong and moderate associations were also observed with GM-CSF and IL-15, respectively (Fig. S6). These robust cytokine data illustrate early activation of T cells in responders. It is encouraging to observe such large differences systemically in a solid tumor setting and suggests changes of the cytokine milieu within the TME.

### Lete-cel promotes remodeling of TME by decreasing macrophage levels

**Fig. 6:**
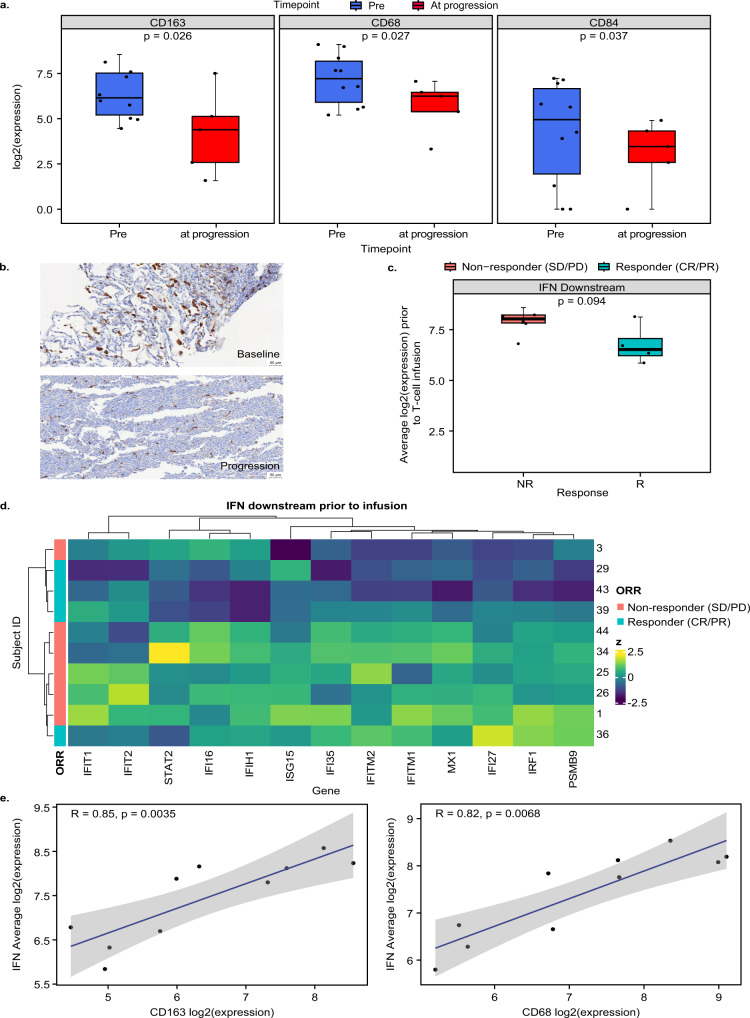
Tumor remodeling post lete-cel infusion. **a** Macrophage markers *CD163, CD68*, and *CD84* pre-infusion (*n* = 10 biologically independent samples) and at progression (*n* = 5 biologically independent samples). **b** CD163 expression in brown by IHC in patient (Subject ID 36) in baseline and at progression biopsy (Day 125) from the same lung lesion (representative region of tissue from an IHC run). **c**, **d** Average expression and heatmap of IFN downstream genes pre-infusion (*n* = 10 biologically independent samples). **e** Association between *CD163* (left) and *CD68* (right) and IFN downstream genes. Line of best fit shown in blue and gray area represents 95% confidence bands. Pre-infusion samples from seven archival screening samples (~1 year pre-infusion) and three fresh baseline samples (pre-lymphodepletion). At progression, samples consist of five samples. Box plots depict median as horizontal line within box, with box bounds as the first and third quartiles. Dots represent individual data points. Lower whisker is the minimum value of the data within 1.5 times the interquartile range below the 25th percentile. Upper whisker is the maximum value of the data within 1.5 times the interquartile range above the 75th percentile. Heatmap show z-scores per gene. Nominal two-sided *p*-values obtained from linear mixed effects models (**a**), limma models (**c**), and standard test for Spearman correlation coefficient (**e**). IFN interferon, IHC immunohistochemistry, NR non-responder, R responder.

While limited tumor biopsies were available in this study (*n* = 15), gene expression analysis revealed interesting trends. Consistent with previous reports^15,16,18,31^, gene expression and immunohistochemistry (IHC) data indicated that SS tumors were primarily infiltrated by CD163 and CD68 macrophages (Fig. S7a, b). Macrophages are associated with poorer prognosis in STS^18,32^. To understand the impact of lete-cel on the TME, we analyzed 10 pre-infusion biopsies and 5 biopsies at progression. Notably, post lete-cel infusion, there was a significant decrease in mRNA expression of macrophage markers *CD163*, *CD68*, and *CD84* (Fig. 6a). The decrease in *CD163* mRNA was confirmed by IHC in all samples (Fig. S7c) and a set of paired samples where a baseline biopsy from the lung had ~7% of the tumor area positive for CD163 compared to ~1% in a biopsy from the same lesion at progression (Day 125) (Fig. 6b). This patient was treated with 1.8 billion cells (0.033 *10^9/kg) and had a partial response with a 55% reduction in tumor size. The growing lesion had continued expression of NY-ESO-1 (100% positive), and the patient received a second infusion ~9 months after the first. Following bridging therapy with trabectedin, the patient received the same LDR and 2 billion cells (0.042 *10^9/kg) from a new product manufacture and had a complete response. Together, these data provide early evidence that lete-cel treatment can not only kill antigen positive tumor cells but also has the potential to remodel the TME, particularly the macrophage component.

This study highlights the negative impact of myeloid cells more broadly on lete-cel treatment. Genes involved in IFN downstream signaling were elevated in non-responders prior to infusion (Fig. 6c, d, Table S5,S6). This gene set is primarily induced by type I interferons, such as IFNα and IFNβ, with limited involvement of IFNγ. Type I interferons are known to play an important role in antiviral response as well as myeloid differentiation^33^. They can have both pro- and antitumor effects depending on the TME, cell types, and cytokines^34–36^. It is unclear what triggered the induction of these genes in this analysis as *IFNA* and *IFNB* RNA was undetectable in tumor cells. However, Fig. 6e shows that the gene set expression was associated with *CD163* and *CD68* macrophage expression. These data suggest a negative association of myeloid cells with lete-cel response and support further investigation of combinations with therapies that target the myeloid compartment.

**Fig. 7:**
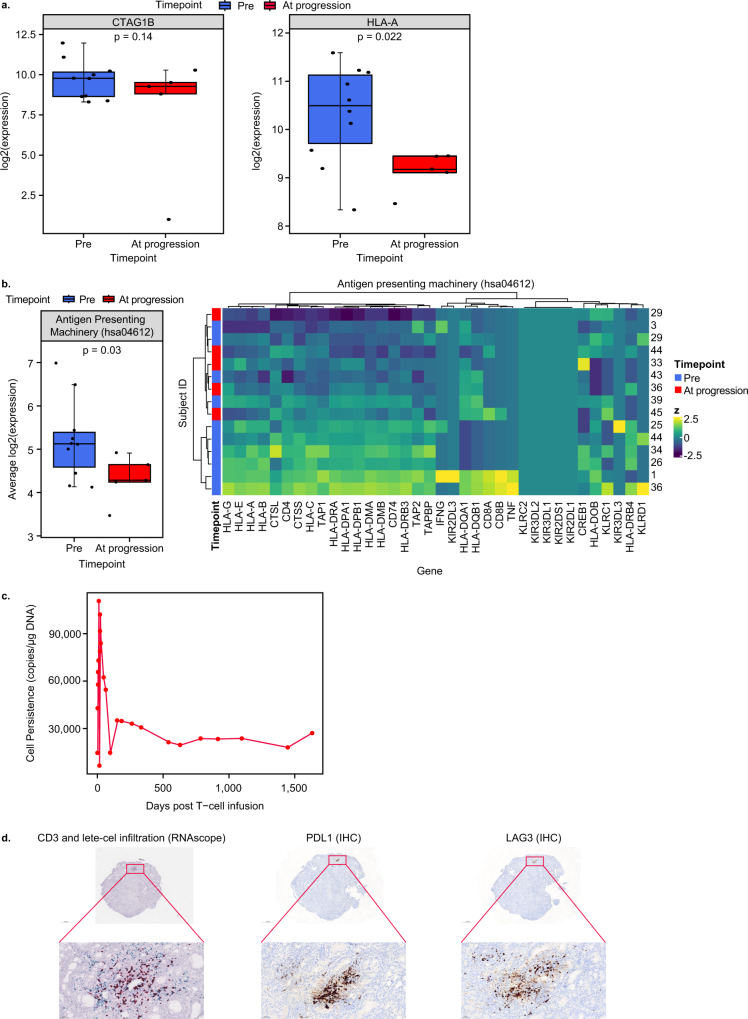
Decreased expression of HLA-A and antigen-presenting genes at progression. **a** Change in gene expression of *CTAG1* (NY-ESO-1) and *HLA-A* between pre-infusion (*n* = 10 biologically independent samples) and at progression (*n* = 5 biologically independent samples). **b** Average expression and heatmap of antigen presentation genes at pre-infusion (*n* = 10 biologically independent samples) and progression (*n* = 5 biologically independent samples). The following genes had background expression across all samples: *KLRC2*, *KIR3DL2*, *KIR3DL1*, *KIR2DS1*, and *KR2DL1*. **c** Persistence of lete-cel in blood of patient 32. **d** Characterization of patient 32 biopsy taken 919 days post-infusion. RNAScope results show CD3 cells in blue and lete-cel in red (left) (representative region of tissue from an IHC run). PDL1 and LAG3 staining in brown by IHC (middle and right images). Tumor samples were primarily from lung metastases. Pre-infusion samples from seven archival screening samples (~1 year pre-infusion) and three fresh baseline samples (pre-lymphodepletion). At progression, samples consist of five samples. Box plots depict median as horizontal line within box, with box bounds as the first and third quartiles. Dots represent individual data points. Lower whisker is the minimum value of the data within 1.5 times the interquartile range below the 25th percentile. Upper whisker is the maximum value of the data within 1.5 times the interquartile range above the 75th percentile. Heatmaps show *z*-scores per gene. Nominal two-sided *p*-values obtained from linear mixed effects models (**a**, **b**). HLA human leukocyte antigen, IHC immunohistochemistry, NR non-responder, R responder.

To evaluate mechanisms of resistance, we analyzed biopsies obtained at progression (range, 95–276 days post-infusion) for *CTAG1B* (NY-ESO-1) and *HLA-A* gene expression. *CTAG1B* RNA expression remained high and was consistent with protein expression (Fig. 7a, S8a). There was a decrease in *HLA-A* and genes involved in antigen presentation at progression (Fig. 7a, b). This could be one means of antigen escape, as HLA-A is necessary for presentation of the NY-ESO-1 peptide to T cells. Availability of paired biopsies limited this analysis, so data are presented for all evaluable samples, but similar trends were observed with three paired biopsies. Further analyses are necessary to understand if this decrease is due to transcriptional downregulation, which could be upregulated in response to IFNγ^35^, or mutations in antigen-presenting genes.

Analysis of genes linked to cancer progression, such as *TGFB* and *WNT* was also performed. As expected, we found high expression of these genes in SS^11,37^. Since expression of these genes was uniformly upregulated, no association with response or PFS was observed (Fig. S8b, c). With limited immune cell infiltration, expression of exhaustion markers was low at all timepoints and had no association with response or PFS (Fig. S8d, e). However, these are bulk RNA analyses which are limited in their ability to characterize rare cell types; single-cell profiling should be conducted in the future to analyze T-cell specific gene signatures.

The need for scRNAseq analysis is highlighted in an interesting case of a responder with evidence of lete-cel tumor infiltration in a biopsy taken 919 days post-infusion and persistence of cells in the blood for >1500 days (Fig. 7c). Further IHC analysis of this sample revealed continued expression of NY-ESO-1 (90% positive) as well as expression of PDL1 and LAG-3 in a similar region of the tissue as lete-cel (Fig. 7d). Future scRNAseq analysis will enable specific characterization of infiltrating T-cells to further our understanding of mechanisms of resistance.

Together these data demonstrate that lete-cel traffic into the tumor and promote tumor remodeling with associated decreases in macrophage levels, offering key insights into considerations for subsequent treatments and combinations of therapies aiming to enhance antitumor effects.

## Discussion

TCR T-cell therapies have shown encouraging clinical results in several solid tumor types^4–9^, including up to 50% overall response rates with lete-cel in SS^7^. However, few biomarker correlates with cell therapy response in solid tumors have been identified. This comprehensive analysis of 45 SS patients treated with lete-cel identified significant associations between LDR, cell dose, and product attributes with response for the first time. These data demonstrate that LDRs containing fludarabine augment IL-15 levels prior to infusion, which is associated with response. Furthermore, standard LDR together with cell dose/kg increases cell expansion and probability of response. Analysis of the product revealed activated, EM cells were associated with response. Finally, tumor analyses post-infusion illustrated lete-cel infiltration into an immune desert tumor and remodeling of the TME by decreasing macrophage levels. Though data are limited by number of patients and available samples, these findings are intriguing and suggest directions for cell dose and LDR optimization, balanced composition of product phenotypes, efficacy enhancement technologies, and combinations of therapies to positively impact clinical response. Further work is required and ongoing to validate these observations in a larger set of patients and paired biopsies.

Many of our findings are consistent with observations of CAR T-cell therapy in hematologic malignances, suggesting similar mechanisms of action. With both cell therapy modalities, peak cell expansion is associated with response^38–40^. We also report a relationship between cell dose and expansion that has been reported for TCR T-cell therapies but is not well defined for CAR T cells^6,27^. Our observations align with reports that LDRs containing a combination of cyclophosphamide and fludarabine led to increased CAR T-cell expansion as well as greater increases in IL-15^8,24^. The association of IL-15 with response supports future exploration of lete-cel in combination with IL-15 agonists. Post-infusion, significant increases in the levels of IFNγ, IL-6, and GM-CSF were observed in responders, suggesting T-cell activation, in line with previous reports^28^.

The association of infused EM CD8 T cells with response is a unique finding for TCR T-cell therapy. The impact of EM cells has been highlighted in the context of response to checkpoint inhibitors^41,42^, but only one study has performed a similar characterization of TCR T-cell product. Nagarsheth, et al. found that HPV-16 E7 TCR T-cell product consisted primarily of EM and TEMRA cells, though there was no association with response and the impact of the number of EM cells infused was not evaluated^6^. Several studies have characterized the memory composition of CAR T-cell products and have underscored the role of stem-like and CM cells and/or genes for durable responses in hematologic malignancies^38,43–45^. These observations suggest different cell populations may play unique roles for immediate versus durable response and CAR T cells versus TCR T cells. Unlike CAR T cells that recognize cell surface antigens, TCR T cells recognize an antigen presented by an HLA molecule, and therefore depend on HLA for target recognition and activation. Interestingly, although HLA expression on the tumor is low in SS^15,19^, we observed responses with lete-cel. Based on our findings, we hypothesize that EM cells are necessary to upregulate HLA expression through IFNγ. EM cells are characterized by higher IFNγ production than CM or stem-like cells^46,47^ and this is further supported in this study by the correlation of CD8 EM cells to peak IFNγ post-infusion (Fig. 3d). Several studies have demonstrated a key role of IFNγ in promoting host-immune recruitment to tumors^30^. Zhang, et al. showed that systemic IFNγ treatment of SS and MRCLS tumors led to increased expression of HLA-ABC which subsequently increased T-cell infiltration^35^. In this study, the ability to confirm HLA upregulation was limited by availability of tumor biopsies collected soon after T-cell infusion. Future studies to characterize the TME within a few weeks post-infusion and gene profiling to better define cell populations in the product will be beneficial. Techniques that link gene expression to traditional cell markers will aid in identifying specific populations of interest; current studies use a mixture, which makes comparison across studies difficult. The enrichment of CD8 EM cells in the apheresis of responders and the associations of infused CD8 EM cells with response, Cmax, and peak IFNγ consistently highlight the importance of this cell type for lete-cel treatment of SS. Further studies are necessary to understand the balance of EM cells, associated with response, and stem-like cells, which may be beneficial for cell persistence.

Treatment of solid tumors with cell therapies poses several challenges such as T-cell trafficking into the tumor and an immune-suppressive TME^48–50^. SS tumors in particular are characterized by a core oncogenic program which is associated with immune cell evasion^51^. Though tumor samples were limited, data illustrated the ability of lete-cel to infiltrate the tumor and promote changes in the immune microenvironment. This is in line with a recent report that CAR T-cells reshape the TME and recruit and activate host-immune cells to the tumor through IFNγ and IL-12^30^. Our TME analyses revealed that elevated IFN response genes, involved in myeloid differentiation and recruitment, were associated with non-responders and that levels of macrophage genes decreased post lete-cel treatment. These findings are consistent with reports of IFN pathway involvement in resistance to CAR T cells^52^ and association of macrophages with poor prognosis in STS^18,32^. Such tumor remodeling could have an important impact on patient response with subsequent treatments.

In terms of mechanisms of resistance, we evaluated antigen loss and T-cell exhaustion^53,54^. Since the SS patients in this study had high expression of NY-ESO-1 at screening, our data showed no association with response. This may differ in tumor types with more heterogenous NY-ESO-1 expression, such as non-small cell lung cancer, gastric, and others. Gene expression analysis revealed no change in NY-ESO-1 expression post-treatment, consistent with previous reports^8^, but a decrease in HLA-A expression and antigen-presenting genes, which may be a mechanism of antigen escape. Nagarsheth et al. reported similar findings of *HLA* and *B2M* mutations in tumor biopsies from three patients who showed no response or relapse to treatment with HPV-16 E7 TCR T cells^6^. Loss of heterozygosity of HLA genes has also been reported as a mechanism of immune evasion in treatment with tumor-infiltrating T-cell therapy^55^. Further analysis is needed to determine if the observed downregulation of HLA in this study is due to transcriptional differences, mutations, or copy number variations. If these are transcriptional differences, strategies to maintain upregulation of HLA over time should be considered. For evaluation of exhaustion markers, this study highlighted limitations of bulk gene expression analysis to characterize tumors with few immune infiltrates and pointed to the benefits of single-cell profiling techniques. Collectively, these data provide unique insights into combinations to further enhance responses: for example, therapies that can decrease the myeloid and macrophage components or upregulate antigen presentation.

This study highlighted the benefits of standard LDR and higher cell dose for robust T-cell expansion, identified product attributes associated with response, and showed a trend in tumor remodeling post T-cell infiltration. Future investigations will include how lete-cel treatment and tumor remodeling may affect the endogenous T-cell repertoire. Our analysis also provided valuable insight into the development of next-generation engineered T-cell technologies, for example data supporting the benefit of CD4 T cells in the product and the high expression of TGF-β in SS tumors. Technologies focused on increasing the persistence of CD4 T cells through co-expression of CD8α to stabilize the HLA interaction, as well as reducing the inhibitory effect of TGF-β on T cells through a dominant negative receptor, are already being evaluated and it will be interesting to monitor impact on efficacy and biomarkers in this study (NCT04526509). Future work will evaluate the strength of these biomarker correlations in disease indications beyond SS, such as MRCLS and non-small-cell lung carcinoma. The findings of our study contribute to better understanding of mechanisms underlying response and resistance to engineered TCR T-cell therapies in SS, a model for solid tumors.

## Methods

### Study design

The study was conducted in accordance with the Declaration of Helsinki and Good Clinical Practice guidelines following approval by the following ethics committees and institutional review boards at each study site: National Institutes of Health Intramural Institutional Review Board, Memorial Sloan Kettering Cancer Center Institutional Review Board/Privacy Board, The University of Texas M.D. Anderson Cancer Center Institutional Review Board, City Of Hope Institutional Review Board, University of South Florida, Research Integrity & Compliance Office Institutional Review Board-Human Research Protection Program, Dana Farber Cancer Center Institutional Review Board, Washington University School of Medicine in Saint Louis, Department Human Research Protection Office, and Office of Human Subject Protection at The Children’s Hospital of Philadelphia. All patients provided written informed consent prior to the performance of any study-specific procedures. The investigator agreed to provide the subject sufficient time to review the document, to inquire about details of the trial, and to decide whether or not to participate in the study. The informed consent was signed and dated by the study subject and by the person who conducted the informed consent discussion. The informed consent for pediatric subjects was signed and dated by the parent or legal guardian of the study subject and by the person who conducted the informed consent discussion. No participant compensation was given except for travel/lodging expenses reimbursement.

This was a multicenter, open-label, pilot study to determine the efficacy and safety of lete-cel in patients with unresectable, metastatic, or recurrent SS. Study stages included screening, leukapheresis/manufacture, lymphodepletion, and treatment phases, followed by long-term follow-up performed under a separate protocol (Fig. S1). Patient were screened for HLA and NY-ESO-1 and considered enrolled at the time of leukapheresis. The total population enrolled (underwent apheresis) and thus considered for the intent to treatment analysis (ITT) was 50 patients (cohort 1: 15, cohort 2: 14, cohort 3: 5, cohort 4:16). A total of five patients dropped out between leukapheresis and T cell infusion. Three patients dropped out due to death (one each from cohort 1, 2, 4), one patient withdrew consent (cohort 1), and one patient withdrew due to disease progression prior to treatment (cohort 1). The modified ITT (mITT) population was 45 patients (cohort 1: 12, cohort 2: 13, cohort 3: 5, cohort 4 :15). The mITT population included 47% (21/45) females and 53% (24/45) males with a median age of 32 years (range of 11–73 years) (Table S1).

### Study participants

#### Inclusion criteria

Patients had to be ≥4 years of age, >18 kg, have Eastern Cooperative Oncology Group (ECOG) performance status of 0–1 (or Lansky >60 for children aged ≤10 years), life expectancy >3 months, and adequate organ function. Additional eligibility requirements were: measurable, pathologically, or cytologically-diagnosed unresectable or metastatic or progressive/persistent or recurrent SS previously treated with and intolerant/non-responsive to a standard chemotherapy regimen containing ifosfamide and/or doxorubicin; NY-ESO-1 positivity, with criteria as detailed for individual cohorts in Table 1; and HLA-A*02:01-, HLA-A*02:05-, and/or HLA-A*02:06-positivity by high-resolution testing.

#### Exclusion criteria

Patients were excluded if they had alanine aminotransferase levels >2.5 times the upper limit of the normal range (ULN) without documented liver metastases/tumor infiltration; total bilirubin >1.5 times ULN (isolated bilirubin >1.5 time ULN was acceptable if bilirubin was fractionated and direct bilirubin was <35%); current active liver or biliary disease; clinically significant systemic illness; untreated CNS metastasis; previous treatment with genetically engineered NY-ESO-1–specific T cells; or a history of active, chronic, or recurrent severe autoimmune or immune-mediated disease requiring steroids or other immunosuppressive treatments.

### Study treatment and procedures

#### Lete-cel manufacture

Autologous T cells were manufactured from patient derived apheresis at either Cell and Vaccine Production Facility at the University of Pennsylvania or at Progenitor Cell Therapy. CD25-depleted CD4+ and CD8 + T cells were activated and expanded using αCD3/αCD28 antibody-conjugated beads (Life Technologies) and then used to develop the engineered T cells. T cells were transduced with a self-inactivating lentivirus vector, derived from HIV-1 containing a woodchuck hepatitis virus posttranscriptional regulatory element, at a multiplicity of infection of 1 transducing unit per cell. T cell manufacturing time ranged from 28 to 35 days, including release testing. Transduction potency was measured on primary T cells.

#### Study procedures

Patients underwent screening assessments, including collection of blood for HLA typing and IHC evaluation of tumor for NY-ESO-1 expression, followed by biological sample collection for laboratory assessments, and collection of disease and medical history (Fig. S1). Eligible patients enrolled to the study underwent apheresis. Patients received infectious prophylaxis for *Pneumocystis carinii*, herpes zoster, and herpes simplex the day prior to commencing lymphodepletion, or as clinically indicated. Prior to lete-cel infusion, patients underwent lymphodepletion dosing as described in Table 1.

On Day 0, patients received thawed lete-cel by intravenous infusion. Patients ≥40 kg received a target dose of 5 × 10^9^ transduced cells, with a minimum 1 × 10^9^ transduced cells and a maximum of 6 × 10^9^ transduced cells. Patients underwent radiologic disease assessments at weeks 4, 8, 12 and every 3 months thereafter, and underwent safety assessments throughout the trial at scheduled timepoints. Patients who progressed are followed for long-term toxicity until death or for up to 15 years post T cell infusion.

Patients could receive a maximum of 2 infusions with lete-cel provided eligibility criteria were met. Patients in any cohort who had a confirmed response or had stable disease for >3 months but then progressed were eligible for a second infusion using the same lymphodepleting regimen as for the first infusion. Patients in Cohorts 3 and 4 who had progressive disease or stable disease as best response for ≤3 months could receive a second infusion using the high dose of fludarabine and cyclophosphamide lymphodepletion regimen. The second infusion could be given no sooner than 60 days from the first infusion and no later than 2 years after the first infusion.

### Study endpoints

#### Efficacy

The primary efficacy outcome was investigator-assessed objective response rate (ORR; complete response or partial response) per Response Evaluation Criteria in Solid Tumors (RECIST) v1.1. This was previously reported for Cohort 1 only by D’Angelo, et al.^7^. Secondary efficacy outcomes included duration of response (DoR), progression-free survival (PFS), best overall response, and overall survival (OS). Best overall response and DoR were previously reported by D’Angelo, et al. for Cohort 1 and Ramachandran, et al. for all cohorts^7,8^. Here we report PFS and OS across all cohorts, as well as overall response rate as assessed by independent review committee.

#### Safety

Secondary safety endpoints included adverse events (AEs), serious AEs (SAEs), and AEs of special interest (AESI); all were evaluated using Common Terminology Criteria for Adverse Events v4.0 (CTCAE v4.0). The safety profile of Cohort 1 was previously reported by D’Angelo, et al.^7^. Here we report on safety events across all cohorts.

#### Biomarkers

Exploratory biomarker endpoints included correlation of expansion, phenotype, and functionality of lete-cel in the blood and or tumor with response to treatment as well as correlation of biomarkers in tumor tissue and blood with response following infusion of lete-cel. The correlation of expansion with response was previously reported by D’Angelo, et al. for Cohort 1^7^. The expansion levels per cohort were summarized by Ramachandran, et al. for all cohorts^8^. Here we report on additional associations of LDR and cell dose with cell expansion, as well as associations of product attributes, cytokines and tumor gene expression with response.

### T-cell expansion and phenotypic analyses

#### T-cell expansion analysis

Peripheral blood mononuclear cell (PBMC) samples were collected and monitored for expansion of gene-modified cells in patients at baseline (7 days prior to chemotherapy); days 4 and 7; weeks 2, 4, and 8; and months 3, 6, and 12 post-infusion. Thereafter, samples were collected every 3 months until 2 years; every 6 months until 5 years; and then every year until 15 years post-infusion. Total DNA was purified using the QIAamp DNA Blood Mini Kit (Qiagen, Hilden, Germany). Presence of the WPRE or Psi transgenes (part of lentiviral vector used to transduce the T cells) was measured with custom Taqman probes by quantitative polymerase chain reaction (qPCR) on ViiA7 Real Time PCR System (Life Technologies, Waltham, MA) at Cambridge Biomedical (now part of BioAgilytix, Boston, MA). Cell kinetics or expansion was determined by quantifying the DNA copy number based on a standard curve consisting of plasmid DNA containing WPRE and Psi sequences.

#### T-cell–phenotypic analysis

PBMC samples were collected at baseline, 1 week; 1, 2, 6, and 12 months; then every 3 months until 2 years post-infusion; and then every 6 months until 5 years post-infusion. Immunophenotyping was performed on cryopreserved PBMC samples using flow cytometry (Caprion, Montreal, Canada; now part of CellCarta).

Detection reagents and concentrations used for T-cell phenotyping Pheno 1 panel are as follows: Live/Dead Fixable Aqua Stain Kit (ThermoFisher, Catalog#:L34957,Dilution: 1/200 Lot#2157152), CD3 (Clone: UCHT1, BD Biosciences, Catalog #: 557943, Dilution: 1/100 Lot#: 9050801), CD4 (Clone: RPA-T4, BD Biosciences,Catalog #: 562658, Dilution 1/50 Lot # 8351537), CD8 (Clone:RPA-T8, BD Biosciences, Catalog #:563821, Dilution 1/800 Lot#: 9073878), CD95 (Clone: DX2, BD Biosciences, Catalog #: 563132, Dilution: 1/100 Lot#: 9003850), CCR7 (Clone: G043H7, BioLegend, Catalog #: 353226, Dilution:1/25 Lot#: B238508), CD127 (Clone: A01D5, BioLegend, Catalog #: 351310, Dilution: 1/100 Lot#: B279332), CD45RO (Clone: UCHL1, BD Biosciences, Catalog #: 560607, Dilution: 1/100 Lot#: 0310596), CD45RA (Clone: 2H4, Beckman Coulter, Catalog #: IM2711U, Dilution: 1/25 Lot#: 200080), CD25 (Clone: M-A251, BD Biosciences, Catalog #: 557753, Dilution: 1/50 Lot#:9345728), LAG-3 (Clone: N/Av, Cedarlane, Catalog #: FAB2319F, Dilution: 2/25 Lot#: ABCB0417081), TIM-3 (Clone: 344823, Cedarlane, Catalog #: FAB2365A, Dilution: 1/25 Lot#: ABFB0417101), PD-1 (Clone: EH12.2H7, BioLegend, Catalog #: 329930, Dilution: 1/50 Lot#: B290009), and NY-ESO-1 Pentamer (HLA-A*0201, ProImmune, Dilution: 1/100 Lot#:TP/7712-21). Detection reagents and concentrations used for T-cell phenotyping Pheno 2 panel are as follows: Live/Dead Fixable Aqua Stain Kit (ThermoFisher, Catalog#:L34957,Dilution: 1/200 Lot#2157152), CD3 (Clone: OKT3, BioLegend, Catalog #: 317328, Dilution: 1/400 Lot#: B289427), CD4 (Clone: RPA-T4, BioLegend, Catalog #:300554, Dilution: 1/50 Lot #B309549), CD8 (Clone:RPA-T8, BD Biosciences, Catalog #:563821, Dilution 1/800 Lot#: 9073878), CD28 (Clone: CD28.2, BD Biosciences, Catalog #: 562976, Dilution: 1/50 Lot#:0265818), CD27 (Clone: O323, Life Technologies, Catalog #: 47-0279-42, Dilution: 1/50 Lot#: 2114191), CD103 (Clone: Ber-ACT8, BD Biosciences, Catalog #: 563883, Dilution: 1/200 Lot#:0247897), CD154/CD40L (Clone: TRAP1, BD Biosciences, Catalog #: 563589, Dilution: 2/25 Lot#:0030198), CD278/ICOS (Clone: DX29, BD Biosciences, Catalog #: 562833, Dilution: 1/50 Lot#:9317172), CD134/OX-40 (Clone: ACT35, BD Biosciences, Catalog #: 563663, Dilution: 1/100 Lot#: 0065008), CD137/4-1BB (Clone: 4B4-1, BioLegend, Catalog #: 309816, Dilution: 2/25 Lot#:B292000), CT152/CTLA-4 (Clone: BNI3, BD Biosciences, Catalog #: 562743, Dilution: 1/50, Lot#: 0030269), CD274/PD-L1 (Clone: MIH1, BD Biosciences, Catalog #: 558065, Dilution: 2/25 Lot#:9143730), and NY-ESO-1 Pentamer (HLA-A*0201, ProImmune, Dilution: 1/100 Lot#:TP/7712-21). See more details in Table S7. PBMCs were thawed (1 × 10^6^ cells/panel) and incubated with Fc blocker (for Pheno2 only) for 10 min at room temperature, prior to being washed and subjected to pentamer staining (10 min at room temperature). PBMCs were then washed and stained with surface stainer (30 min at 4 °C), then washed again and fixed in 0.5% paraformaldehyde (30 min at 4 °C). Cells were then washed again before acquisition. Data was acquired on LSR II flow cytometer (BD Biosciences) and analyzed using FlowJo software (BD). Gating strategy is shown in Fig. S9. Hierarchical gating was used for all markers except stem-cell memory cells, which used Boolean gating (CD45RA + CCR7 + CD45RO-CD95 + CD127+).

### Serum cytokine analysis

Concentration of serum cytokines was measured at baseline; day 0 pre-infusion; 1, 4, and 7 days post-infusion; and 2, 3, 4, 6, and 8 weeks post-infusion by the Meso Scale Discovery (MSD) immunoassay at Cambridge Biomedical (Boston, MA; now part of BioAgilytix). Three commercially available kits (V-PLEX Proinflammatory Panel 1; V-PLEX Cytokine Panel 1 and U-PLEX Biomarker Group 1) were used to collectively analyze the cytokine profiles of GM-CSF, IFN-γ, IL1α, IL1β, IL-1RA, IL-2, IL-2Rα, IL-4, IL-5, IL-6, IL-7, IL-8, IL-10, IL-12/IL-23p40, IL-12p70, IL-13, IL-15, IL-16, IL-17A, TNF-α, TNF-β and VEGF-A. Data was collected using the MESO QuickPlex SQ120 (MSD).

### Analyses of tumor biopsies

#### RNA analysis

RNA analysis was performed by Histogenex (Wilrijk, Belgium; now part of CellCarta). For each sample, five 4 µm unstained slides were used for macrodissection and subsequent RNA extraction. RNA extract was quantified (including assessment of RNA purity) using the Quant-iT RiboGreen RNA Reagent and Kit, and RNA quality was assessed using Agilent RNA Pico chip analysis.

RNA was analyzed using the NanoString nCounter® system, with 2 sets of NanoString assays (nCounter® PanCancer Immune Profiling Panel and nCounter® PanCancer Pathway Panel) run on the same extract. The normalization for raw nCounter counts of expressed genes is separately done for QC-passed samples in PanCancer immune and PanCancer pathway panel using the R-package NanoStringNorm (R version 4.0.3) with a set of parameters; CodeCount = ‘geo.mean’, Background = ‘mean.2sd’, SampleContent = ‘housekeeping.geo.mean’, round.values = TRUE, take.log = TRUE.

#### Detection of TCR T cells by RNA in situ hybridization

RNA in situ hybridization on biopsied tissues was performed using the RNAscope® 2.5 HD Duplex Reagent Kit (Advanced Cell Diagnostics, Newark, CA) comprising TCR T cell and CD3 mRNA detection assays. 5-μm formalin-fixed, paraffin-embedded (FFPE) tissue sections were pretreated with heat and protease prior to hybridization with the target oligo probes. Preamplifier, amplifier, and horseradish peroxidase/alkaline phosphatase-labeled oligos were then hybridized sequentially, followed by chromogenic precipitate development.

Each sample was quality controlled for RNA integrity with an RNAscope® probe specific to PPIB/POLR2A RNA and for background with a probe specific to bacterial dapB RNA. The RNAscope® CD3 probe comprised a pool of three human CD3 antigens (CD3d, CD3e, and CD3g, Advanced Cell Diagnostics, Catalog #: 426628) mRNA, whereas the TCR probe was custom-made. Specific RNA staining signal was identified as green or red punctate dots. Samples were counterstained with hematoxylin. Representative images were digitally obtained using CaseViewer (3D Histech, Budapest, Hungary).

#### Protein expression by IHC

NY-ESO-1 staining was performed using the E978 clone (Sigma, Catalog #: N2038, at 1 μg/mL) at QualTek Laboratory (Goleta, CA; now part of Discovery Life Sciences). The following markers were analyzed at Histogenex (Wilrijk, Belgium; now part of CellCarta) by IHC (all analyzed using Ventana Benchmark XT unless noted otherwise): CD4 (clone SP35, Ventana, catalog #: 790–4423, no dilution), CD8 (clone C8/144B, Dako, catalog #: M7103, 1/75 dilution), CD20 (clone L26, Ventana, catalog #: 760–2531, no dilution), CD45 (clone 2B11 + PD7/26, Agilent; catalog #: M070101, 1/100 dilution, staining on autostainer), CD163 (clone MRQ-26, Ventana, catalog #: 760–4437, no dilution), LAG-3 (clone 17B4, Novus biologicals, catalog #:97657, 1/2000 dilution), Pan Keratin (clone AE1/AE3/PCK16, Ventana, catalog #: 760–2595, no dilution), PD-1/CD279 (clone SP269, Abcam, catalog #: GR3208557-2, 1/50 dilution), PD-L1 (clone SP142, Ventana, catalog #: M4424, 1/250 dilution), and TIM-3 (clone D5D5R, Cell Signaling Technologies, catalog #: 45208, 1/250 dilution). Representative images were digitally obtained using CaseViewer (3D Histech, Budapest, Hungary).

### Statistical analysis

#### Populations for analysis

The intent-to-treat (ITT) population included all enrolled patients, whereas the modified ITT (mITT) population, used for safety and efficacy assessments, included patients who received lete-cel infusion. The population for biomarker analyses included patients who received lete-cel infusion with available biomarker data; sample size for each population is specified in figure legends.

#### Endpoint analyses

The primary endpoint for the study was pre-defined as objective response (ORR) using RECIST v1.1 based on Investigator assessment. A sensitivity analyses by Independent Review was also assessed. The ORR for each cohort of the mITT population was evaluated using 95% Clopper-Pearson confidence intervals.

Secondary efficacy endpoints included best overall response (BoR), duration of response (DoR), progression-free survival (PFS) and overall survival (OS) were evaluated using descriptive statistics and Kaplan–Meier plots as appropriate. PFS was defined as the time from T-cell infusion to the earliest documentation of disease progression or death from any cause or surgical resection or start of prohibited medication. OS was defined as the time from t-cell infusion to death due to any cause. For this analysis data from the parent study and the long-term follow-up study were included up to the data cut-off of April 23, 2021. Subjects known to be alive at this time were censored for the OS analysis.

Exploratory biomarker endpoints were evaluated as specified below. As these were exploratory endpoints, the study was not powered to evaluate these assessments. *P*-values are presented for descriptive purposes and are nominal (unadjusted) unless noted otherwise.

#### Evaluation of expansion correlates

Post-hoc relationships between cell expansion, biomarker expression, and efficacy were evaluated in a hypothesis-driven manner using Wilcoxon, logistic, linear, and median regression (R version 3.5.1), after log-transforming data when appropriate.

#### Flow cytometry analysis

For flow cytometry analysis, all samples with <5000 viable CD3 + cells in either the Pheno1 or Pheno2 panel were removed in downstream analysis. When the frequency for the Pentamer+ parent gate for a sample was lower than the noise level determined from the maximum value for negative control samples (healthy donors in which there should not be any Pentamer+ cells), we applied a flooring by setting the frequency of the parent gates to 0 and subsequent children gates to ‘not available’ for all samples. This flooring was separately done for each CD8 + Pentamer and CD4 + Pentamer+ populations. To calculate the number of transduced cells for memory phenotypes, we first calculated the percentage of CD4 + cells among Pentamer+ cells (%CD4 + Pentamer+) using the counts of CD3 + CD4 + Pentamer+ and CD3 + CD8 + Pentamer+ cells in product as %CD4 + Pentamer+ = (number of CD3 + CD4 + Pentamer+)/((number of CD3 + CD4 + Pentamer+) + (number of CD3 + CD8 + Pentamer+)). Accordingly, the percentage of CD8 + cells among Pentamer+ cells (%CD8 + Pentamer+) was inferred as 1 – %CD4 + Pentamer+. Then, the number of transduced cells of memory phenotype *X* for each sample was inferred as (number of CD4 + Pentamer+ *X* phenotype) = (%CD4 + Pentamer+) × (number of transduced cells in sample) × %CD4 + Pentamer+*X*+. The number of transduced cells of CD8 + Pentamer+ memory phenotype *X* was done similarly. *P*-values in downstream statistical analyses were based on the Wilcoxon rank sum test, whereas correlations were based on Spearman correlation coefficients.

#### Cytokine analysis

Cytokine analysis was based on a linear mixed effects model, log-transformed cytokine level modeled with treatment x time interactions and a random subject intercept (with otherwise independent errors). For cytokines having ≤1 left-censored (below lower limit of quantification) value, the R-package lme4 (version 1.1–23) was used. For cytokines having ≥2 left-censored values, left censoring was addressed using the R-package lmec^56^ (version 1.0). In all cases Kenward & Roger^57^ degrees-of-freedom were used, calculated for lmec models using the R-package pbkrtest (version 0.4–8.6). Continuous AR1 models were also fit for cytokines having ≤1 left-censored value; although they often resulted in better BIC values, they did not change substantive conclusions. As lmec does not support continuous AR-1 models, independence models were kept for all cytokines. Analyses were conducted in R version 4.0.2.

#### RNA analysis

For RNA analysis, both gene-level and gene-set analyses were conducted for Nanostring data, with each of the two panels fit separately. ORR comparisons were based on limma models^58^ and implemented via the limma R/Bioconductor package (version 3.44.3), with log2-expression as response and ORR as a single covariate. Pre-treatment versus At-Progression comparisons were conducted using linear mixed effects models (lme4, version 1.1–23) to address repeated measures.

PFS comparisons were based on Cox proportional hazards models with PFS as response and log2- expression as a single covariate. To summarize differential expression across gene sets, global significance statistics were based on t-statistics from each gene within a set of interest ($\surd \sum {t}^{2}$). Corresponding *p*-values were obtained by permutation test (5000 permutations). Similarly, Competitive Fisher test odds ratios were based on cross-tabulation of gene set membership with nominal significance (*p* < 0.05); *p*-values were obtained by permutation test using Fisher Exact test *p*-value as objective function. Gene sets were obtained by selecting collections from KEGG (all HSA sets), GO (immune and cellular communication processes), REACTOME (all HSA sets), MsigDB (C4, C6, and Hallmarks), and a few other hand-curated sets obtained from Nanostring files or literature review^59,60^; only gene sets with ≥5 overlapping non-constant genes and ≥50% coverage of the original gene set on the respective panel were considered. Analyses were conducted in R version 4.0.2.

### Reporting summary

Further information on research design is available in the Nature Research Reporting Summary linked to this article.

## Supplementary information

Supplementary information

Reporting Summary

## Data Availability

For reasons of privacy protection for study participants, GSK offers access to data and materials via controlled access. Anonymized individual participant data from this study plus the annotated case report form, protocol, reporting and analysis plan, dataset specifications, raw dataset, analysis-ready dataset, and clinical study report are available for research proposals approved by an independent review committee. Proposals should be submitted to www.clinicalstudydatarequest.com. Responses typically take within 30–45 days for the initial feasibility check. A data access agreement will be required. The data access agreement contains the terms under which GSK will provide access to researchers and institutions to GSK’s clinical data. Data access recipients will be required to handle the data in accordance with data protection laws and have appropriate information security systems in place. The agreement also requires that the results of the research conducted using GSK’s data must subsequently be published, either in a scientific journal or pre-print option, and that any software or models developed in the research must be released with open-source access. The RNA gene expression data discussed in this publication have been deposited in NCBI’s Gene Expression Omnibus^61^ and are accessible through GEO Series accession number GSE202981.

## Code availability

Quantification and statistical analyses for all data types was conducted using publicly available packages in R.
