# Supplementary material for: Biomarker correlates with response to NY-ESO-1 TCR T cells in patients with synovial sarcoma
Source: Nat Commun. 2022 Sep 8;13:5296. doi: 10.1038/s41467-022-32491-x (PMC9458750; doi:10.1038/s41467-022-32491-x)
Supplement: Supplementary file 1 — Supplementary information [file 41467_2022_32491_MOESM1_ESM.pdf]

Supplementary information for Biomarkers correlates with response to NY-ESO-1 TCR T cells in patients with synovial sarcoma

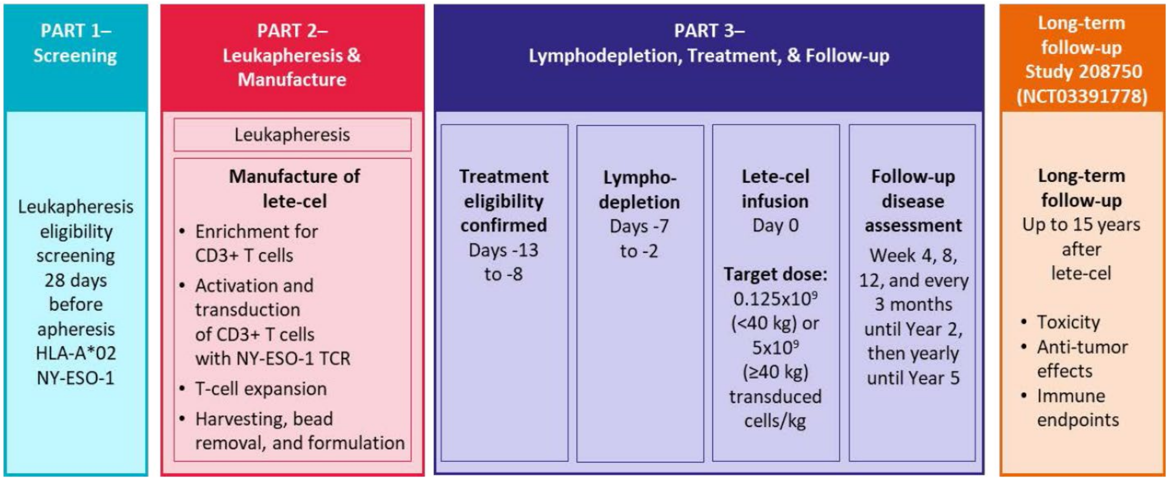

Figure S1: Study design. Overview of study design.

CD3, cluster of differentiation 3; HLA, human leukocyte antigen; lete-cel, letetresgene autoleucel; NY-ESO-1, New York esophageal squamous cell carcinoma 1; TCR, T-cell receptor.

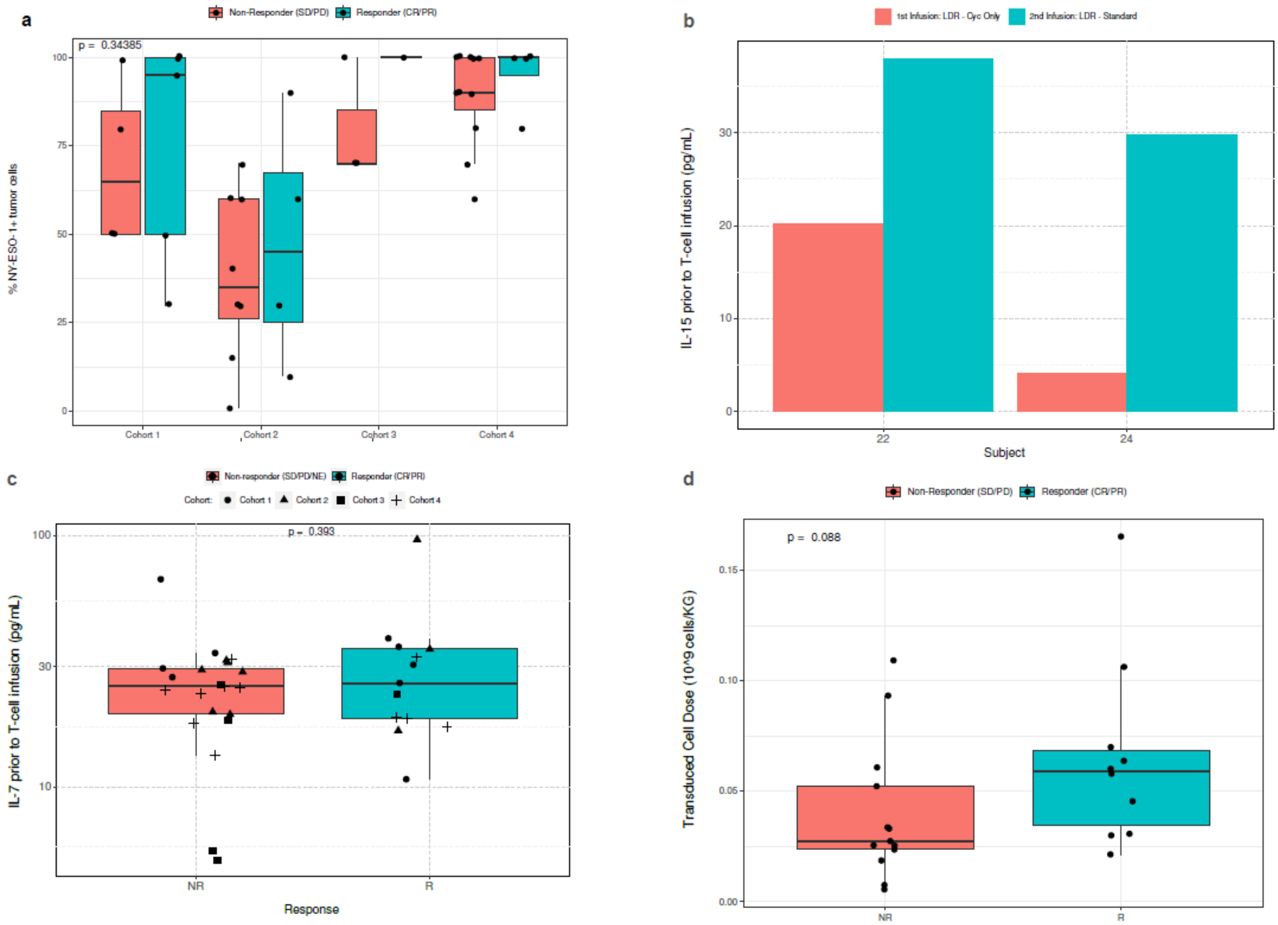

Figure S2: Expression of NY-ESO-1 and cytokines prior to infusion and T-cell dose. a, Expression of NY-ESO-1 at screening across 4 cohorts as measured by IHC with any intensity staining (n=40 biologically independent samples). b, IL-15 levels prior to 1st and 2nd infusion in 2 patients from Cohort 3 which received 2 different LDR prior to each infusion. c, IL-7 levels prior to T-cell infusion across cohorts (n=34 biologically independent samples). d, Impact of transduced cell dose/kg on response (n=19 biologically independent samples).

Box plots depict median as horizontal line within box, with box bounds as the first and third quartiles. Dots represent individual data points. Lower whisker is the minimum value of the data within 1.5 times the interquartile range below the 25th percentile. Upper whisker is the maximum value of the data within 1.5 times the interquartile range above the 75th percentile. Two-sided p-values were calculated via bootstrapped median regression (10,000 bootstraps) to adjust for cohort or responder status (a),t-test (c), and Wilcoxon test (d). T-test was performed in (c) to be consistent with remaining cytokine analyses; Wilcoxon p-value=0.576. Cyc, cyclophosphamide; IHC, immunohistochemistry; IL, interleukin; LDR, lymphodepleting chemotherapy regimen; NY-ESO-1, New York esophageal squamous cell carcinoma 1.

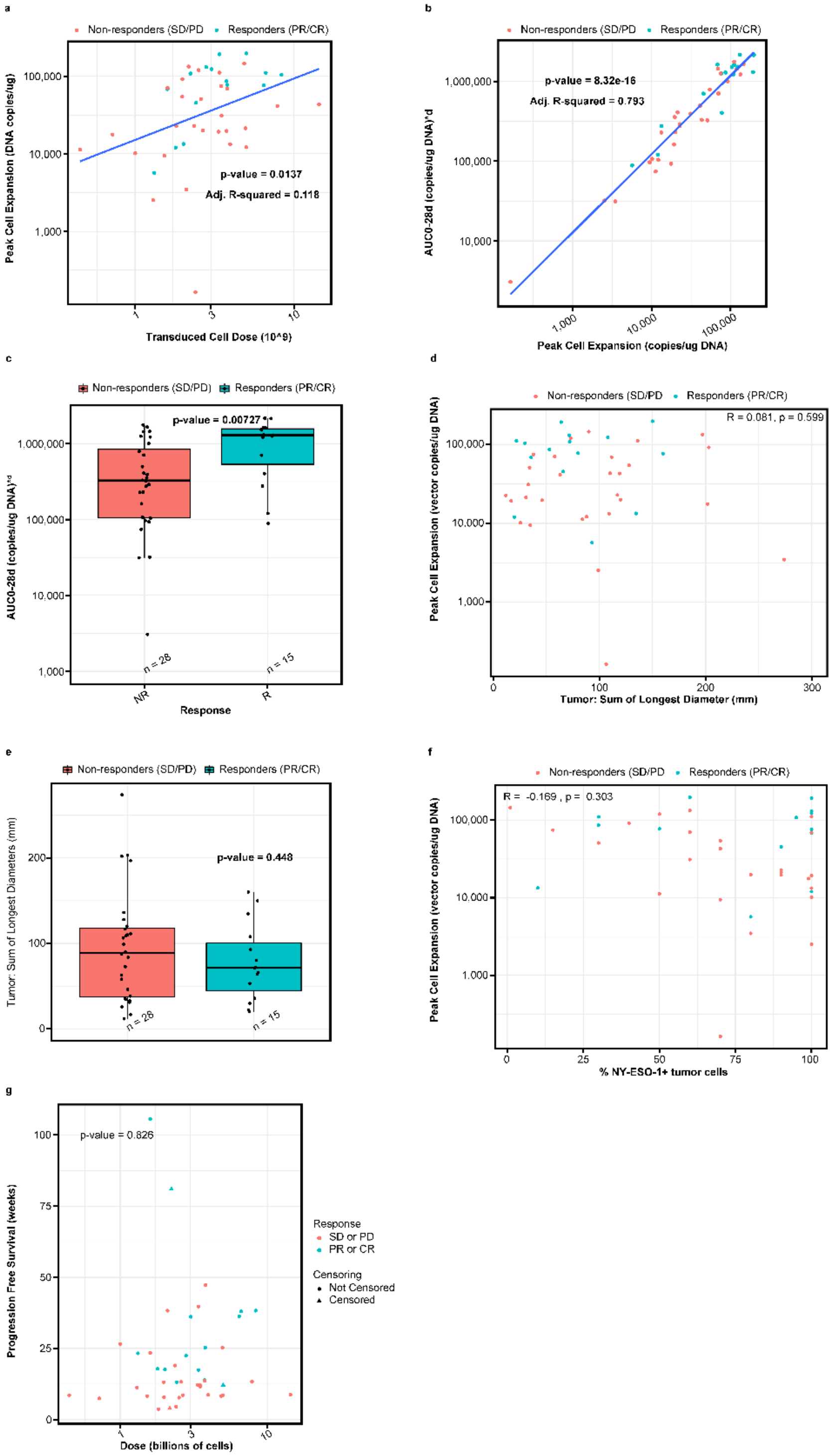

Figure S3: Further characterization of impact of peak cell expansion on response, PFS, and factors that impact expansion. a, Association of cell dose with peak cell expansion. b, Association of AUC0_28 with peak cell expansion. c, Association of AUC0_28 with response. d,e, Relationship of baseline tumor size as measured by sum of longest diameters (mm) and peak cell expansion (d) and response (e). a-e show data from 43 patients. f, Relationship of NY-ESO-1 expression at screening and peak cell expansion (n=40 biologically independent samples). g, Relationship of dose with PFS (n=43 biologically independent samples).

Box plots depict median as horizontal line within box, with box bounds as the first and third quartiles. Dots represent individual data points. Lower whisker is the minimum value of the data within 1.5 times the interquartile range below the 25th percentile. Upper whisker is the maximum value of the data within 1.5 times the interquartile range above the 75th percentile. Two-sided p-values were calculated via linear regression (a, b), Wilcoxon test (c,e), and Cox proportional hazards model (g). AUC0_28, Area under the cell expansion curve from day 0 to day 28; CR, complete response; LDR, lymphodepleting chemotherapy regimen; NY-ESO-1, New York esophageal squamous cell carcinoma 1; PD, progressive disease; PFS, progression-free survival; PR, partial response; SD, stable disease.

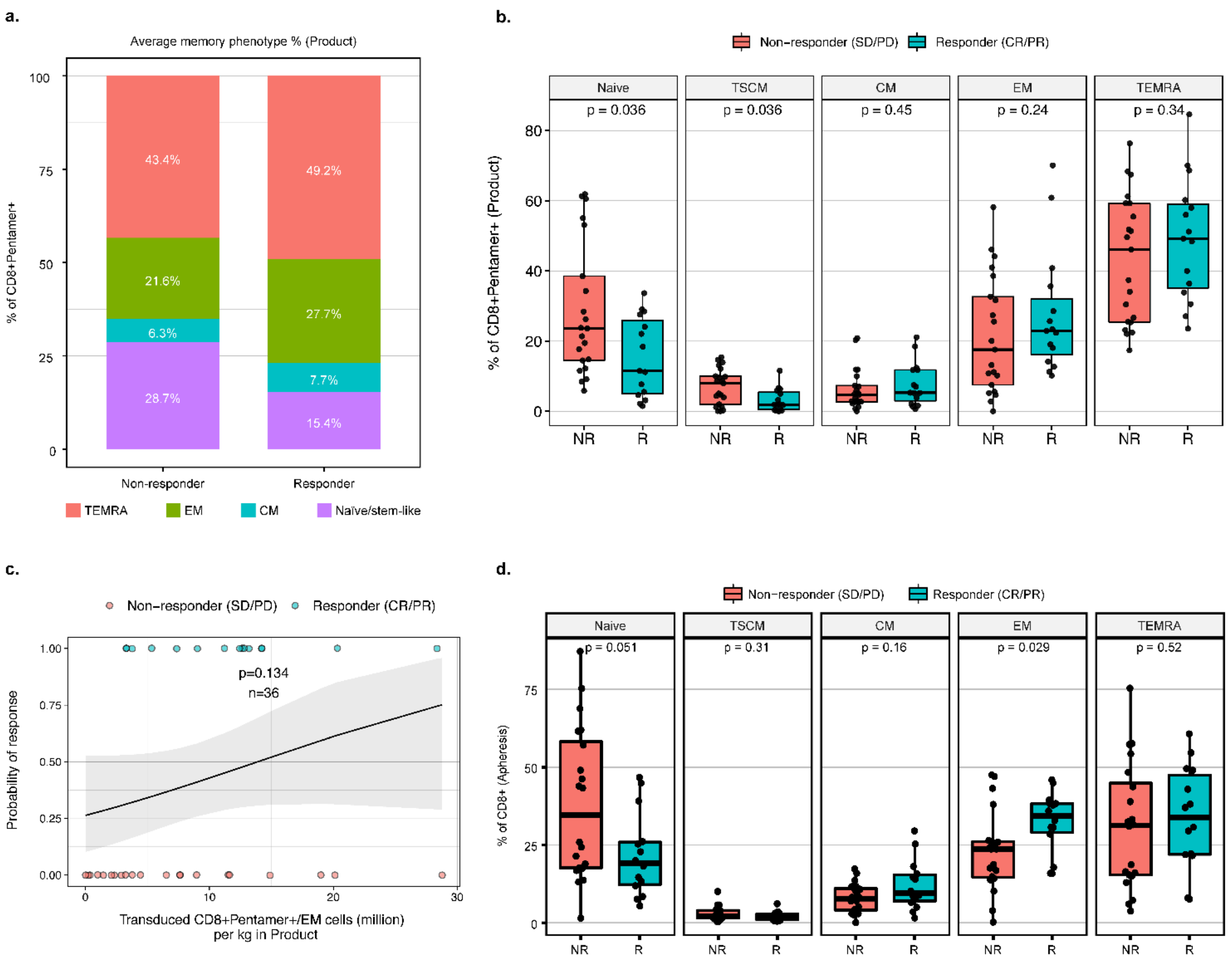

Figure S4: Memory phenotype distribution in T-cell product and apheresis. a, Memory phenotype distribution (a) of CD8+Pentamer+ cells in product of non-responders vs responders. b, Frequency of memory phenotypes in CD8+Pentamer+ cells and association with response (n=36 biologically independent samples). c, Logistic regression in black showing probability of response with number of infused CD8+ Pentamer+ EM cells/kg, grey areas showing 95% confidence bands. d, Relationship of percent memory phenotypes in apheresis with response (n=34 biologically independent samples). Product analyses consist of 36 patients. Apheresis analyses consist of 34 patients.

Box plots depict median as horizontal line within box, with box bounds as the first and third quartiles. Dots represent individual data points. Lower whisker is the minimum value of the data within 1.5 times the interquartile range below the 25th percentile. Upper whisker is the maximum value of the data within 1.5 times the interquartile range above the 75th percentile. Nominal two-sided p-values based on the Wilcoxon rank sum test and correlations are based on Spearman method. CM, central memory (CD45RA-CCR7+); CR, complete response; EM, effector memory (CD45RA-CCR7-); Naïve (CD45RA+CCR7+); PD, progressive disease; PR, partial response; SD, stable disease; TEMRA, T effector memory RA (CD45RA+CCR7-); TSCM, T stem cell memory (CD45RA+CCR7+CD45RO-CD95+CD127+).

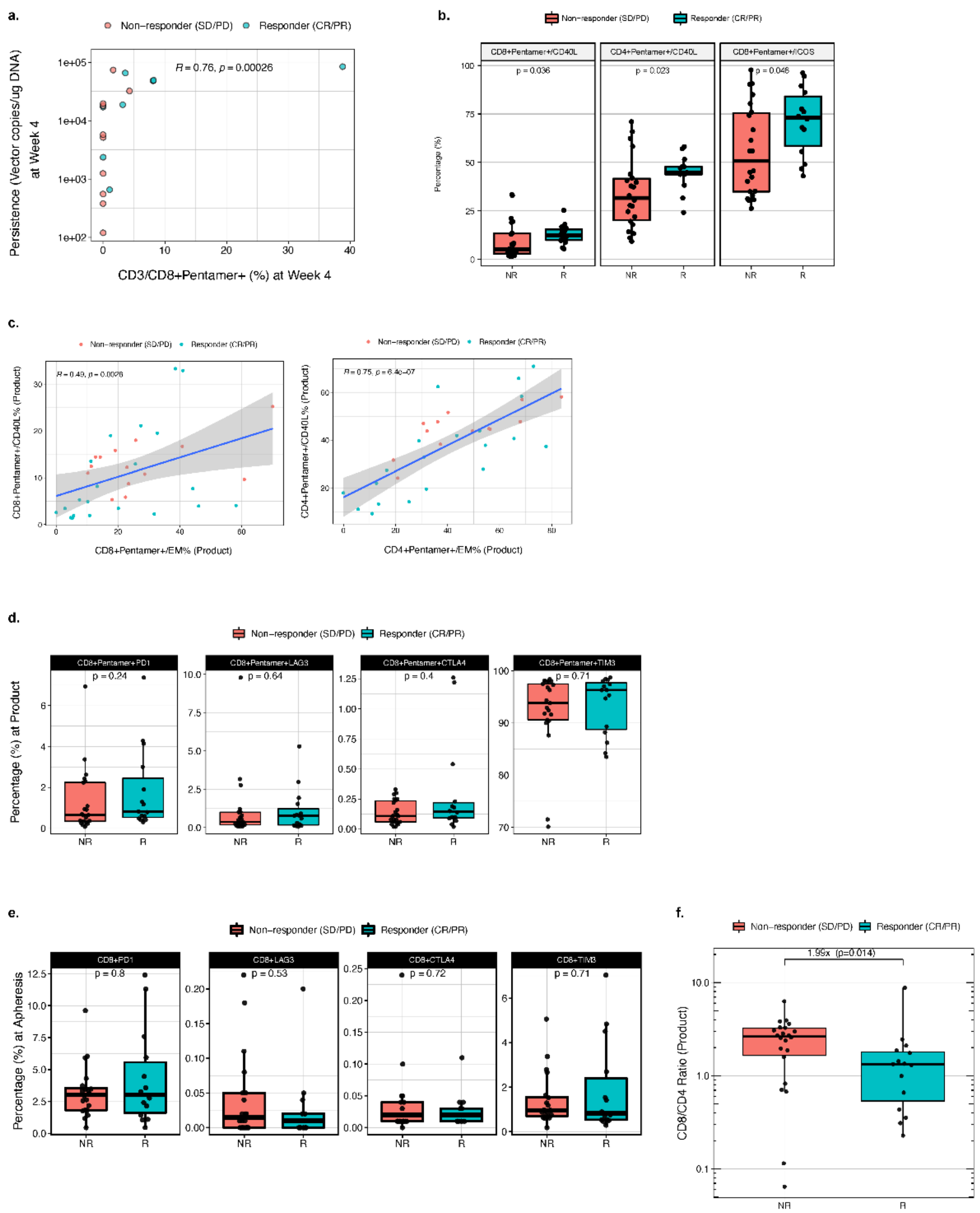

Figure S5: Further characterization of product attributes and associations with response. a, Association of CD8+Pentamer+ cells and persistence at Week 4 (n=18 biologically independent samples). b, Expression of CD40L and ICOS in product (n=36 biologically independent samples). c, Association of CD40L with EM phenotype in CD8+Pentamer+ cells (left) and CD4+Pentamer+ cells (right) in product (n=36 biologically independent samples). Lines of best fit are shown in blue with grey areas showing 95% confidence bands. d,e, Expression of PD-1, LAG3, CTLA4, and TIM3 in CD8+Pentamer+ cells in product(d, n=36 biologically independent samples) and apheresis (e, n=34 biologically independent samples). f, Ratio of total CD8 to CD4 cells in product (n=36 biologically independent samples).

Box plots depict median as horizontal line within box, with box bounds as the first and third quartiles. Dots represent individual data points. Lower whisker is the minimum value of the data within 1.5 times the interquartile range below the 25th percentile. Upper whisker is the maximum value of the data within 1.5 times the interquartile range above the 75th percentile. Nominal two-sided p-values based on the Wilcoxon rank sum test and correlations are based on Spearman method. CM, central memory (CD45RA-CCR7+); CR, complete response; EM, effector memory (CD45RA-CCR7-); Naïve (CD45RA+CCR7+); PD, progressive disease; PR, partial response; SD, stable disease; TEMRA, T effector memory RA (CD45RA+CCR7-); TSCM, T stem cell memory (CD45RA+CCR7+CD45RO-CD95+CD127+).

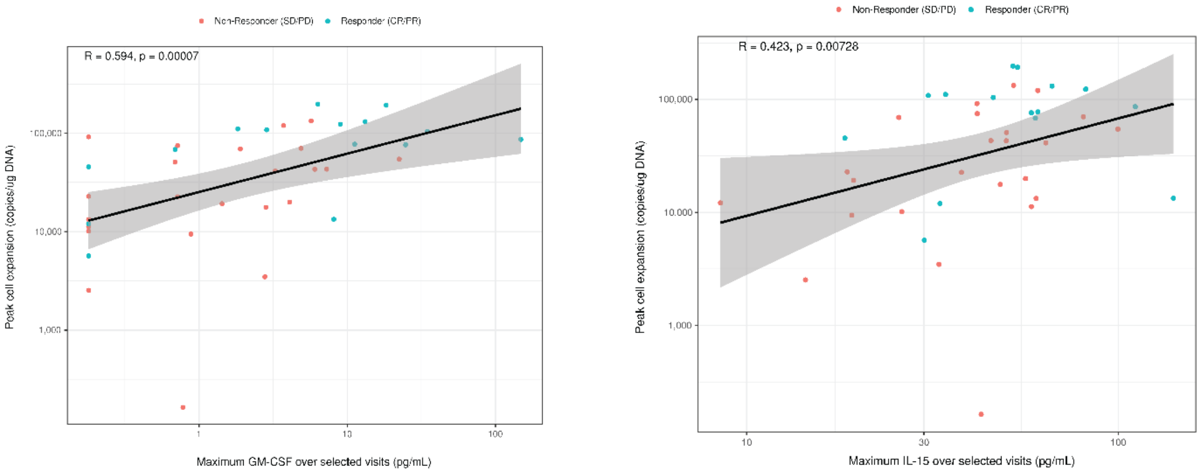

Figure S6: Relationship of cytokines with peak cell expansion. Association of GM-CSF (left) and IL-15 (right) with peak cell expansion (n=39 biologically independent samples).

Lines of best fit are shown in black with grey areas showing 95% confidence bands. Two-sided p-values were calculated via standard test for Spearman correlation. GM-CSF, granulocyte-macrophage colony-stimulating factor; IL, interleukin.

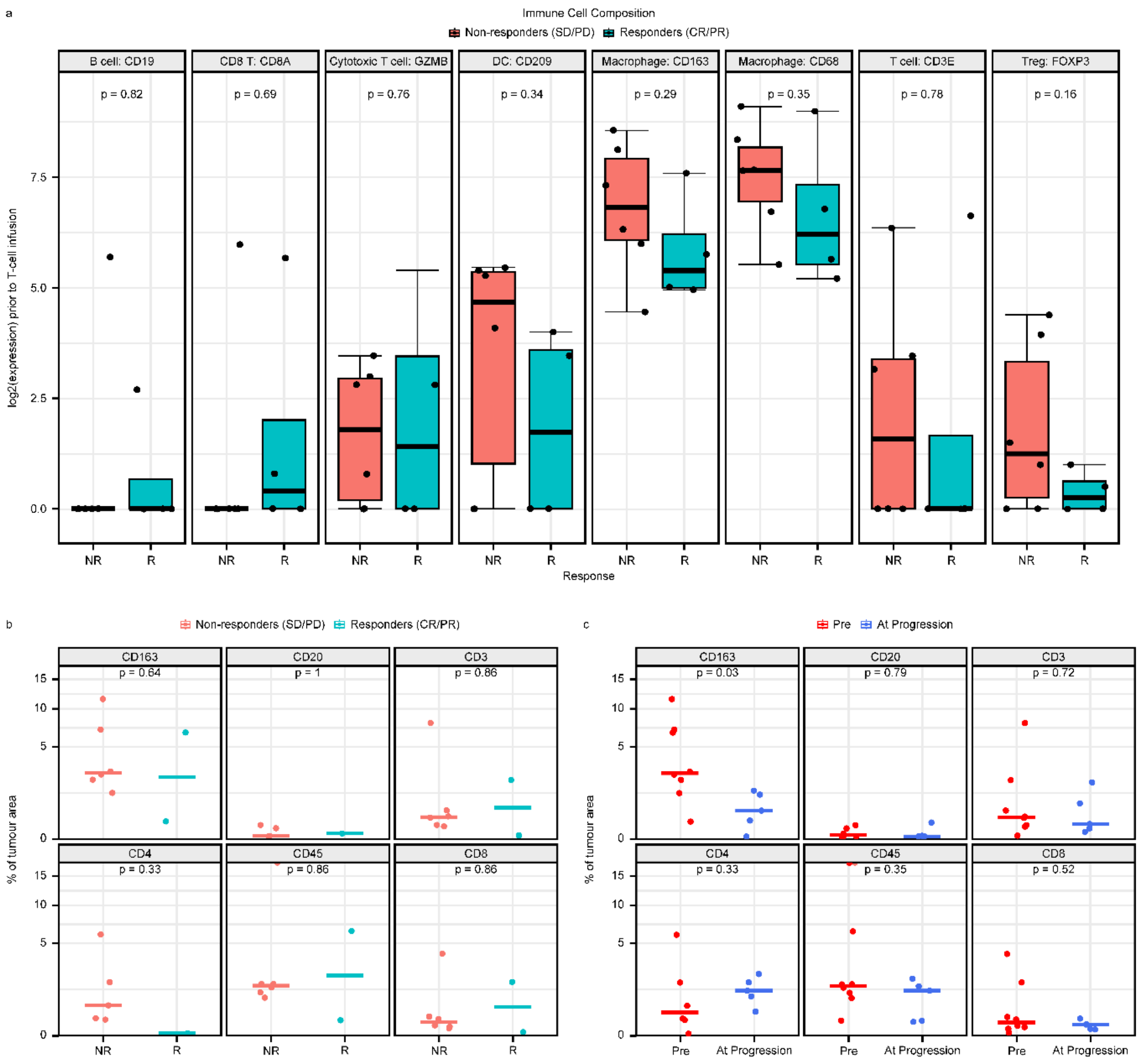

Figure S7: Immune landscape of SS tumors. a, Immune cell composition prior to infusion by gene expression analysis (n=10 biologically independent samples). Box plots depict median as horizontal line within box, with box bounds as the first and third quartiles. Dots represent individual data points. Lower whisker is the minimum value of the data within 1.5 times the interquartile range below the 25th percentile. Upper whisker is the maximum value of the data within 1.5 times the interquartile range above the 75th percentile. b, Immune cell composition prior to infusion by IHC (n=8 biologically independent samples). c, Changes in immune cell composition pre T-cell infusion (n=8 biologically independent samples) and at-progression (n=5 biologically independent samples) by IHC.

Cross-bars indicate median. Nominal two-sided p-values obtained from limma models (a) and Wilcoxon test (b,c). IHC, immunohistochemistry; NR, non-responder; R, responder.

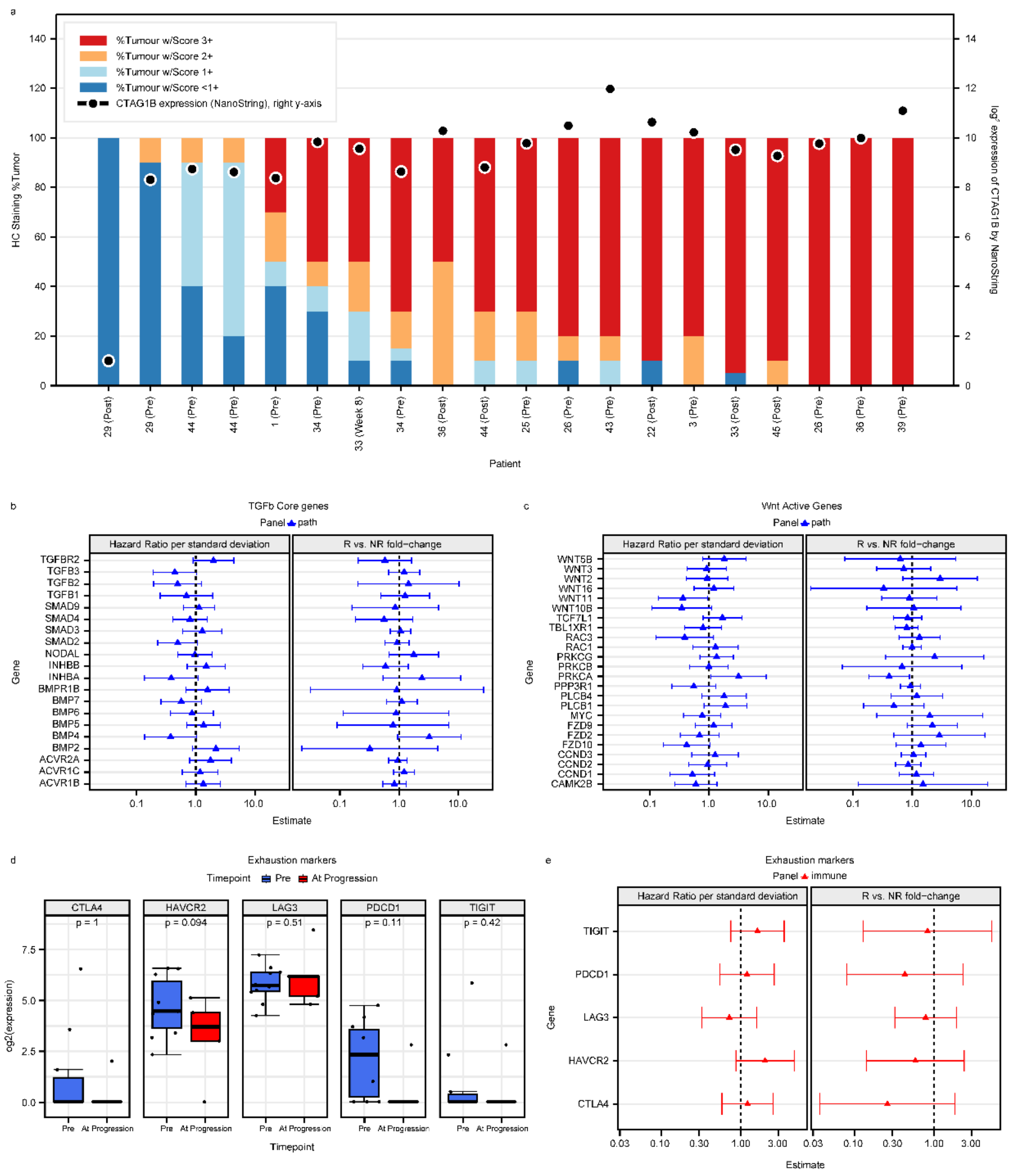

Figure S8: Further characterization of tumor gene analyses. a, Comparison of expression of NY-ESO-1 by IHC and mRNA levels in available tumor biopsies pre or post treatment (n=20). b-c, Differential expression of TGF-β core genes(b) and Wnt activating genes (c) prior to infusion between responders and non-responders and hazard ratio for PFS (n=10 biologically independent samples). d, Change in gene expression of exhaustion markers (HAVCR2 and PDCD1 encode TIM-3 and PD-1 respectively) between pre-infusion (n=10 biologically independent samples) and at progression (n=5 biologically independent samples). Nominal two-sided p-values obtained from linear mixed effects model. e, Differential expression of exhaustion markers prior to infusion between responders and non-responders and hazard ratio for PFS (n=10 biologically independent samples). Pre-infusion samples from 7 archival screening samples (~1 year pre-infusion) and 3 fresh baseline samples (pre-lymphodepletion). At progression, samples consist of 5 samples.

Box plots depict median as horizontal line within box, with box bounds as the first and third quartiles. Dots represent individual data points. Lower whisker is the minimum value of the data within 1.5 times the interquartile range below the 25th percentile. Upper whisker is the maximum value of the data within 1.5 times the interquartile range above the 75th percentile. For b,c,e estimates and 95% confidence intervals from Cox proportional hazards model (left panels) and limma (right panels of R vs NR). IHC, immunohistochemistry; NR, no response; NY-ESO-1, New York esophageal squamous cell carcinoma 1; PFS, progression-free survival; R, response.

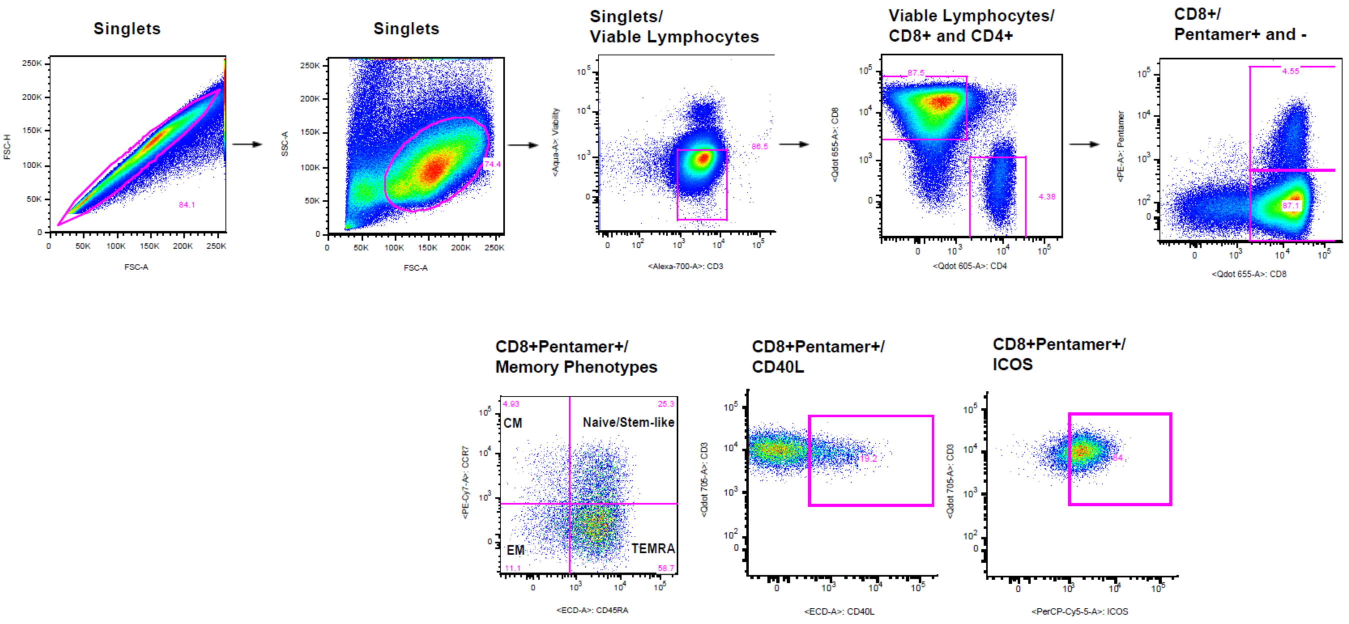

Figure S9: Gating strategy for flow cytometry analysis.

Cells were gated on singlets and viable lymphocytes (CD3+), followed by CD8+ T cells and CD8+/Pentamer+ cells. CD8+Pentmer+cells were then gated on either memory phenotypes, CD40L, or ICOS expression (bottom row) as an example. Data from control transduced TCR T cells is shown as an example. T stem cell memory (Tscm) gating was performed via Boolean gating (CD45RA+CCR7+CD45RO-CD95+CD127+) so is not shown here. CD3, cluster of differentiation 3; CM, central memory (CD45RA-CCR7+); EM, effector memory (CD45RA-CCR7-); Naïve (CD45RA+CCR7+); TEMRA, T effector memory RA (CD45RA+CCR7-).

Table S1: Patient characteristics (mITT population)

Parameter	Cohort 1[a] (n=12)	Cohort 2[a] (n=13)	Cohort 3[a] (n=5)	Cohort 4[a] (n=15)	Overall[a,e] (N=45)
**Sex, n (%)**					
Men	6 (50)	7 (54)	3 (60)	8 (53)	24 (53)
Women	6 (50)	6 (46)	2 (40)	7 (47)	21 (47)
**Median age (range), y[b]**	30 (18–50)	29 (11–73)	25 (15–39)	36 (20–69)	32 (11–73)
**Race, n (%)**					
Black/African American	0	0	1 (20)	1 (7)	2 (4)
White	11 (92)	10 (77)	4 (80)	14 (93)	39 (87)
Other	1 (8)	3 (23)	0	0	4 (9)
**HLA-A status, n (%)**					
HLA-A*02:01	10 (83)	13 (100)	5 (100)	14 (93)	42 (93)
HLA-A*02:05	0	0	0	1 (7)	1 (2)
HLA-A*02:06	1 (8)	0	0	0	1 (2)
Other[c]	1 (8)	0	0	0	1 (2)
**ECOG performance status, n (%)[d]**					
0	7 (58)	6 (46)	4 (80)	8 (53)	25 (56)
1	5 (42)	6 (46)	1 (20)	7 (47)	19 (42)
**Prior systemic therapy, n (%)**	12 (100)	13 (100)	5 (100)	15 (100)	45 (100)
**Bridging therapy, n (%)**					
Yes	0	8 (62)	2 (40)	5 (33)	15 (33)
No	12 (100)	5 (39)	3 (60)	10 (67)	30 (67)

[a]Totals may not sum to 100% due to rounding. [b]At informed consent. [c]Result was ambiguous and assumed to be HLA-A*02:01-positive at >99.99% probability. [d]One subject in Cohort 2 had an ECOG status of 1 at screening but was a status 2 at the time of baseline. Eligibility did not specify subjects must meet ECOG at screening and baseline. [e]Overall column gives sum of all cohorts unless otherwise specified. ECOG, Eastern Cooperative Oncology Group; HLA, human leukocyte antigen; ITT, intention to treat; mITT, modified intention to treat.

Table S2: Incidence of adverse events with toxicity grade ≥3 occurring in ≥10% of subjects

Preferred Term, n (%)	Cohort 1 (N=12)	Cohort 2 (N=13)	Cohort 3 (N=5)	Cohort 4 (N=15)	Overall (N=45)
**Any Adverse Event**	**12 (100.0)**	**13 (100.0)**	**5 (100.0)**	**14 (93.3)**	**44 (97.8)**
Leukopenia/White blood cell decreased	11 (91.7)	12 (92.3)	5 (100.0)	11 (73.3)	39 (86.7)
Neutropenia/Neutrophil count decreased	10 (83.3)	11 (84.6)	4 (80.0)	10 (66.7)	35 (77.8)
Anemia/Red blood cell count decreased	10 (83.3)	11 (84.6)	3 (60.0)	7 (46.7)	31 (68.9)
Thrombocytopenia/Platelet count decreased	8 (66.7)	11 (84.6)	4 (80.0)	7 (46.7)	30 (66.7)
Lymphopenia/Lymphocyte count decreased	12 (100.0)	8 (61.5)	3 (60.0)	6 (40.0)	29 (64.4)
Hypophosphatemia	9 (75.0)	5 (38.5)	2 (40.0)	3 (20.0)	19 (42.2)
Febrile neutropenia	4 (33.3)	4 (30.8)	0	6 (40.0)	14 (31.1)
Dyspnea	1 (8.3)	3 (23.1)	3 (60.0)	0	7 (15.6)
Hyponatremia	3 (25.0)	3 (23.1)	1 (20.0)	0	7 (15.6)
Hypotension	0	3 (23.1)	1 (20.0)	1 (6.7)	5 (11.1)
Rash/Rash maculo-papular	2 (16.7)	2 (15.4)	1 (20.0)	0	5 (11.1)

Table S3: Incidence of serious adverse events occurring in 2 or more subjects

Preferred Term, n (%)	Cohort 1 (N=12)	Cohort 2 (N=13)	Cohort 3 (N=5)	Cohort 4 (N=15)	Overall (N=45)
**Any Serious Adverse Event**	**6 (50.0)**	**7 (53.8)**	**4 (80.0)**	**6 (40.0)**	**23 (51.1)**
Pyrexia	4 (33.3)	1 (7.7)	0	3 (20.0)	8 (17.8)
Cytokine Release Syndrome (CRS)	1 (8.3)	3 (23.1)	0	1 (6.7)	5 (11.1)
Dyspnea	2 (16.7)	1 (7.7)	2 (40.0)	0	5 (11.1)
Febrile neutropenia	2 (16.7)	1 (7.7)	0	2 (13.3)	5 (11.1)
Hypotension	0	3 (23.1)	0	0	3 (6.7)
Neutropenia/Neutrophil count decreased	1 (8.3)	2 (15.4)	0	0	3 (6.7)
Acute kidney injury	1 (8.3)	0	0	1 (6.7)	2 (4.4)
Bone marrow failure	1 (8.3)	1 (7.7)	0	0	2 (4.4)
Chills	2 (16.7)	0	0	0	2 (4.4)
Diarrhea	0	2 (15.4)	0	0	2 (4.4)
Guillain-Barre syndrome	0	2 (15.4)	0	0	2 (4.4)
Pleural effusion	0	1 (7.7)	1 (20.0)	0	2 (4.4)
Rash/Rash maculo-papular	2 (16.7)	0	0	0	2 (4.4)
Thrombocytopenia/Platelet count decreased	1 (8.3)	1 (7.7)	0	0	2 (4.4)

Table S4: Characteristics and response for subset of patients that received two infusions

Cohort	Subject ID	First Infusion LDR	First infusion Transduced Cell Dose (*10^9)	First Infusion Best Overall Response	Maximal Reduction in Tumor Size (SLD) after 1st infusion	Cmax after 1st infusion	Day for 2nd infusion	Second infusion LDR	Second infusion Transduced Cell Dose (*10^9)	Second Infusion Best Overall Response	Maximal Reduction in Tumor Size (SLD) after 2nd infusion	Cmax after 2nd infusion
1	49	Standard	8.328	CR	-100.0	104114.6	435	Standard	0.49	SD	0.0	14039.4
1	11	Standard	6.45	PR	-65.6	76184.8	387	Standard	1.18	SD	-28.3	46345.6
2	45	Standard	2.42	PR	-51.5	45430.1	177	Standard	2.88	SD	-8.6	43439.0
3	24	Cyc Only	5	SD	+5.7	12139.1	272	Standard[a]	3.4	SD	-8.4	101816.2
3	22	Cyc Only	3.02	PR	-77.8	123313.8	324	Standard	2.02	SD	-88.4	237095.0
4	25	Reduced	3.40	SD	-24.3	111259.9	563	Standard	5	SD	-16.9	62463.4
4	27	Reduced	3.8	SD	-26.5	69256.8	428	Standard	2.79	SD	-31.5	117909.2
4	33	Reduced	2.1	SD	-3.3	3471.3	367	Reduced[b]	1.31	SD	+4.4	48026.9
4	8	Reduced	1.00	SD	+1.2	10147.3	260	Standard	2.2	PR	-65.2	185802.5
4	36	Reduced	1.8	PR	-55	11993.5	272	Reduced	2	CR	-100.0	128247.9
4	26	Reduced	4.95	SD	-19.4	21349.0	289	Standard	4.99	SD	-32.4	341497.7

[a]Reduced fludarabine 20mg/m^2. [b]Reduced fludarabine to 20mg/m^2 and no cyclophosphamide on Day -5

Table S5: Gene sets differentially expressed between Responders and Non-responders prior to infusion from Nanostring Immune panel

R vs. NR_Immune panel			
**Gene set**	**Compet. Fisher p value**	**Nano Global p value**	**Description**
R-HSA-918233	0.0044	0.0196	TRAF3-dependent IRF activation pathway
R-HSA-933541	0.0044	0.0302	TRAF6 mediated IRF7 activation
R-HSA-933543	0.0044	0.0426	NF-kB activation through FADD/RIP-1 pathway mediated by caspase-8 and -10
Nanostring_Immune panel	0.0144	0.0702	IFN Downstream
Nanostring_Immune panel	0.018	0.0958	Microglial Functions
R-HSA-168928	0.0044	0.0976	DDX58/IFIH1-mediated induction of interferon-alpha/beta
GO:0002687	0.111	0.1028	Positive regulation of leukocyte migration
GO:0034128	0.1094	0.1066	Negative regulation of MyD88-independent toll-like receptor signaling pathway
R-HSA-933542	0.0154	0.1092	TRAF6 mediated NF-kB activation
R-HSA-5357786	0.0508	0.1152	TNFR1-induced proapoptotic signaling

NR, non-responder; R, responder. Nominal two-sided p-values.

Table S6: Gene sets differentially expressed between Responders and Non-responders prior to infusion from Nanostring cancer pathway panel

R vs. NR_Cancer pathway panel			
**Gene set**	**Compet. Fisher p value**	**Nano Global p value**	**Description**
R-HSA-1170546	3.388972274	0.0312	Prolactin receptor signaling
R-HSA-450341	3.957244894	0.0366	Activation of the AP-1 family of transcription factors
R-HSA-5621575	2.147246631	0.0618	CD209 (DC-SIGN) signaling
R-HSA-5654719	3.474271689	0.0632	SHC-mediated cascade:FGFR4
R-HSA-5654712	3.474271689	0.0684	FRS-mediated FGFR4 signaling
R-HSA-190322	6.116486546	0.0688	FGFR4 ligand binding and activation
R-HSA-5654228	6.116486546	0.0688	Phospholipase C-mediated cascade; FGFR4
R-HSA-9010642	5.940565516	0.0954	ROBO receptors bind AKAP5
R-HSA-2179392	3.388972274	0.0968	EGFR Transactivation by Gastrin
R-HSA-5654720	4.062699455	0.106	PI-3K cascade:FGFR4

NR, non-responder; R, responder. Nominal two-sided p-values.

Table S7: Detection reagents used for T-cell phenotyping, (A) Panel 1 composition (Pheno1) and (B) Panel 2 composition (Pheno2)

(A)

Marker	Fluorochrome	Clone	Catalog #	Supplier
CD3	Alexa Fluor 700	UCHT1	557943	BD Biosciences
CD4	BV605	RPA-T4	562658	BD Biosciences
CD8	BV650	RPA-T8	563821	BD Biosciences
CD95	BV711	DX2	563132	BD Biosciences
CCR7	PE-Cy7	G043H7	353226	BioLegend
CD127	BV421	A01D5	351310	BioLegend
CD45RO	PerCP-Cy5.5	UCHL1	560607	BD Biosciences
CD45RA	ECD	2H4	IM2711U	Beckman Coulter
CD25	APC-Cy7	M-A251	557753	BD Biosciences
LAG-3	FITC	N/Av	FAB2319F	Cedarlane
TIM-3	APC	344823	FAB2365A	Cedarlane
PD-1	BV785	EH12.2H7	329930	BioLegend
Pentamer*	PE	HLA-A*0201	F049-2A-D*<br>F008-2A-D*	F008-2A-D
Live/Dead Fix Aqua	Aqua	N/A	L34957	Thermo Fisher Scientific

(B)

Marker	Fluorochrome	Clone	Catalog #	Supplier
CD3	BV711	OKT3	317328	BioLegend
CD4	BV785	RPA-T4	300554	BioLegend
CD8	BV650	RPA-T8	563821	BD Biosciences
CD28	BV605	CD28.2	562976	BD Biosciences
CD27	APC-eFluor780	O323	47-0279-42	Life Technologies
CD103	APC	Ber-ACT8	563883	BD Biosciences
CD154 (CD40L)	PE-CF594	TRAP1	563589	BD Biosciences
C278 (ICOS)	PerCP-Cy5.5	DX29	562833	BD Biosciences
CD134 (OX-40)	PE-Cy7	ACT35	563663	BD Biosciences
CD137 (4-1BB)	Alexa Fluor 700	4B4-1	309816	BioLegend
CD152 (CTLA-4)	BV421	BNI3	562743	BD Biosciences
CD274 (PD-L1)	FITC	MIH1	558065	BD Biosciences
Pentamer*	PE	HLA-A*0201	F049-2A-D*<br>F008-2A-D*	ProImmune
Live/Dead Fix Aqua	Aqua	N/A	L34957	Thermo Fisher Scientific

*CMV or NY-ESO-1 pentamer
