# Supplementary material for: Biomarker correlates with response to NY-ESO-1 TCR T cells in patients with synovial sarcoma
Source: Nat Commun. 2022 Sep 8;13:5296. doi: 10.1038/s41467-022-32491-x (PMC9458750; doi:10.1038/s41467-022-32491-x)
Supplement: Supplementary file 2 — Reporting Summary [file 41467_2022_32491_MOESM2_ESM.pdf]

nature portfolio

Corresponding author(s): Ioanna Eleftheriadou PhD

Last updated by author(s): Jul 29, 2022

Reporting Summary

Nature Portfolio wishes to improve the reproducibility of the work that we publish. This form provides structure for consistency and transparency in reporting. For further information on Nature Portfolio policies, see our Editorial Policies and the Editorial Policy Checklist.

Statistics

For all statistical analyses, confirm that the following items are present in the figure legend, table legend, main text, or Methods section.

n/a	Confirmed	
☐	☒	The exact sample size (*n*) for each experimental group/condition, given as a discrete number and unit of measurement
☐	☒	A statement on whether measurements were taken from distinct samples or whether the same sample was measured repeatedly
☐	☒	The statistical test(s) used AND whether they are one- or two-sided<br>*Only common tests should be described solely by name; describe more complex techniques in the Methods section.*
☐	☒	A description of all covariates tested
☐	☒	A description of any assumptions or corrections, such as tests of normality and adjustment for multiple comparisons
☐	☒	A full description of the statistical parameters including central tendency (e.g. means) or other basic estimates (e.g. regression coefficient) AND variation (e.g. standard deviation) or associated estimates of uncertainty (e.g. confidence intervals)
☐	☒	For null hypothesis testing, the test statistic (e.g. *F*, *t*, *r*) with confidence intervals, effect sizes, degrees of freedom and *P* value noted<br>*Give P values as exact values whenever suitable.*
☒	☐	For Bayesian analysis, information on the choice of priors and Markov chain Monte Carlo settings
☒	☐	For hierarchical and complex designs, identification of the appropriate level for tests and full reporting of outcomes
☐	☒	Estimates of effect sizes (e.g. Cohen's *d*, Pearson's *r*), indicating how they were calculated

Our web collection on statistics for biologists contains articles on many of the points above.

Software and code

Policy information about availability of computer code

Data collection	For cell expansion, data was collected using ViiA 7 Real Time PCR system (Life Technologies). For flow cytometry, data was acquired on LSR II flow cytometer (BD Biosciences). Cytokine data was collected using the MESO QuickPlex SQ 120 (MSD). Gene expression was analyzed using the nCounter platform (Nanostring). Representative IHC and ISH images were digitally imaged using CaseViewer v2.3.2 (3D Histech).
Data analysis	Clinical outcome analyses for primary and secondary endpoints were performed using SAS v9.4.<br><br>For flow cytometry, raw data files (.fcs) were analyzed using FlowJo software v9.9(BD). Quantification and statistical analyses for all data types was conducted using publicly available packages in R(v3.5.1 for cell expansion, v4.0.3 for flow cytometry, and v4.0.2 for all other analyses). All available from CRAN or Bioconductor.

For manuscripts utilizing custom algorithms or software that are central to the research but not yet described in published literature, software must be made available to editors and reviewers. We strongly encourage code deposition in a community repository (e.g. GitHub). See the Nature Portfolio guidelines for submitting code & software for further information.

Data

Policy information about availability of data

All manuscripts must include a data availability statement. This statement should provide the following information, where applicable:

- Accession codes, unique identifiers, or web links for publicly available datasets
- A description of any restrictions on data availability
- For clinical datasets or third party data, please ensure that the statement adheres to our policy

For reasons of privacy protection for study participants, GSK offers access to data and materials via controlled access. Anonymized individual participant data from

this study plus the annotated case report form, protocol, reporting and analysis plan, data set specifications, raw dataset, analysis-ready dataset, and clinical study report are available for research proposals approved by an independent review committee. Proposals should be submitted to www.clinicalstudydatarequest.com. Responses typically take within 30-45 days for the initial feasibility check. A data access agreement will be required. The data access agreement contains the terms under which GSK will provide access to researchers and institutions to GSK's clinical data. Data access recipients will be required to handle the data in accordance with data protection laws and have appropriate information security systems in place. The agreement also requires that the results of the research conducted using GSK's data must subsequently be published, either in a scientific journal or pre-print option, and that any software or models developed in the research must be released with open-source access.
The RNA gene expression data discussed in this publication have been deposited in NCBI's Gene Expression Omnibus and are accessible through GEO Series accession number GSE202981 (https://www.ncbi.nlm.nih.gov/geo/query/acc.cgi?acc=GSE202981).

Field-specific reporting

Please select the one below that is the best fit for your research. If you are not sure, read the appropriate sections before making your selection.

☒ Life sciences ☐ Behavioural & social sciences ☐ Ecological, evolutionary & environmental sciences

For a reference copy of the document with all sections, see nature.com/documents/nr-reporting-summary-flat.pdf

Life sciences study design

All studies must disclose on these points even when the disclosure is negative.

Sample size

This trial was originally designed as a single cohort, single arm pilot study to determine whether T cells genetically engineered to recognize an HLA-A2 binding peptide from NY-ESO-1 induce antitumor responses in subjects with synovial sarcoma. The trial expansion was based on favorable results from the single arm pilot study of subjects that formed Cohort 1. The trial was amended to enroll further subjects into three additional cohorts designated as Cohorts 2, 3 and 4. This study was not powered for formal hypothesis testing. For each cohort, a minimum of 5 subjects were treated to enable the interim analysis. At interim analysis, predictive probabilities based on observed response rate together with the safety and other study data were used to determine whether to stop the cohort for futility or to continue enrollment to further confirm safety and efficacy signal observed. Clinical efficacy is presented on modified intent to treat population, patients who received lete-cel infusion (n=45).

The population for biomarker analyses included patients who received lete-cel infusion with available biomarker data; sample size for each population is specified in figure legends. This study was not powered for formal hypothesis testing of biomarker associations.

Data exclusions

For flow cytometry analysis, samples with <5,000 viable CD3+ cells were excluded to ensure robust starting population for analysis.
For gene expression analysis, two samples were excluded due to low RNA concentration.

Replication

Samples were run in triplicate for cell expansion and duplicate for cytokines to ensure reproducibility; all attempts at replications were successful. Flow cytometry and tumor analyses (gene expression and IHC experiments) could not be replicated due to limited sample availability.

Randomization

This was a non-randomized study. Cohort 1 was enrolled first and the study then expanded to include Cohorts 2,3, and 4. Patients were allocated to Cohorts 2,3, and 4 based on NY-ESO-1 expression and slot availability for each cohort. Cohort 2 was enrolled in parallel with Cohorts 3 and 4, while Cohorts 3 and 4 accrued sequentially.

Blinding

Blinding was not relevant since this was a pilot study and all correlative analyses were exploratory.

Reporting for specific materials, systems and methods

We require information from authors about some types of materials, experimental systems and methods used in many studies. Here, indicate whether each material, system or method listed is relevant to your study. If you are not sure if a list item applies to your research, read the appropriate section before selecting a response.

Materials & experimental systems

n/a	Involved in the study	
☐	☒	Antibodies
☒	☐	Eukaryotic cell lines
☒	☐	Palaeontology and archaeology
☒	☐	Animals and other organisms
☐	☒	Human research participants
☐	☒	Clinical data
☒	☐	Dual use research of concern

Methods

n/a	Involved in the study	
☒	☐	ChIP-seq
☐	☒	Flow cytometry
☒	☐	MRI-based neuroimaging

Antibodies

Antibodies used	For flow cytometry: CD3 (Clone: UCHT1, BD Biosciences, Catalog #: 557943, Dilution: 1/100 Lot#: 9050801); CD3 (Clone: OKT3, BioLegend, Catalog #: 317328, Dilution: 1/400 Lot#: B289427); CD4 (Clone: RPA-T4, BD Biosciences,Catalog #: 562658, Dilution 1/50 Lot # 8351537); CD4 (Clone: RPA-T4, BioLegend, Catalog #:300554, Dilution: 1/50 Lot #B309549); CD8 (Clone:RPA-T8, BD Biosciences, Catalog #:563821, Dilution 1/800 Lot#: 9073878); CD95 (Clone: DX2, BD Biosciences, Catalog #: 563132, Dilution: 1/100 Lot#: 9003850); CCR7 (Clone: G043H7, BioLegend, Catalog #: 353226, Dilution:1/25 Lot#: B238508); CD127 (Clone: A01D5, BioLegend, Catalog #: 351310, Dilution: 1/100 Lot#: B279332); CD45RO (Clone: UCHL1, BD Biosciences, Catalog #: 560607, Dilution: 1/100 Lot#: 0310596); CD45RA (Clone: 2H4, Beckman Coulter, Catalog #: IM2711U, Dilution: 1/25 Lot#: 200080); CD25 (Clone: M-A251, BD Biosciences, Catalog #: 557753, Dilution: 1/50 Lot#:9345728); LAG-3 (Clone: N/Av, Cedarlane, Catalog #: FAB2319F, Dilution: 2/25 Lot#: ABCB0417081); TIM-3 (Clone: 344823, Cedarlane, Catalog #: FAB2365A, Dilution: 1/25 Lot#: ABFB0417101); PD-1 (Clone: EH12.2H7, BioLegend, Catalog #: 329930, Dilution: 1/50 Lot#: B290009); NY-ESO-1 Pentamer (HLA-A*0201, ProImmune, Dilution: 1/100 Lot#:TP/7712-21); CD28 (Clone: CD28.2, BD Biosciences, Catalog #: 562976, Dilution: 1/50 Lot#:0265818); CD27 (Clone: O323, Life Technologies, Catalog #: 47-0279-42, Dilution: 1/50 Lot#: 2114191); CD103 (Clone: Ber-ACT8, BD Biosciences, Catalog #: 563883, Dilution: 1/200 Lot#:0247897); CD154/CD40L (Clone: TRAP1, BD Biosciences, Catalog #: 563589, Dilution: 2/25 Lot#:0030198); CD278/ICOS (Clone: DX29, BD Biosciences, Catalog #: 562833, Dilution: 1/50 Lot#:9317172); CD134/OX-40 (Clone: ACT35, BD Biosciences, Catalog #: 563663, Dilution: 1/100 Lot#: 0065008); CD137/4-1BB (Clone: 4B4-1, BioLegend, Catalog #: 309816, Dilution: 2/25 Lot#:B292000); CT152/CTLA-4 (Clone: BNI3, BD Biosciences, Catalog #: 562743, Dilution: 1/50, Lot#: 0030269); CD274/PD-L1 (Clone: MIH1, BD Biosciences, Catalog #: 558065, Dilution: 2/25 Lot#:9143730)<br><br>For IHC: NY-ESO-1 (Clone: E978, Sigma, Catalog #: N2038, Dilution to 1ug/mL); CD4 (Clone: SP35, Ventana, Cat #: 790-4423,No dilution); CD8 (Clone: C8/144B, Dako, Catalog #: M7103, Dilution: 1/75); CD20 (Clone: L26, Ventana, Catalog #: 760-2531, No dilution); CD45 (Clone: 2B11 + PD7/26,Agilent, Catalog #: M070101, Dilution 1/100), CD163 (Clone: MRQ-26, Ventana, Catalog #: 760-4437, No dilution), LAG-3 (Clone: 17B4, Novus biologicals, Catalog #: 97657, Dilution 1/2000), PanCK (Clone: AE1/AE3/PCK16, Ventana, Catalog #: 760-2595, No Dilution), PD-1/CD279 (Clone: SP269,Abcam, Catalog #:GR3208557-2, Dilution 1/50), PD-L1 (Clone: SP142, Ventana, Catalog #: M4424, Dilution 1/250), and TIM-3 (Clone: D5D5R, Cell Signaling Technologies, Catalog #: 45208 , Dilution 1/250)
Validation	See manufacturer's product information below for flow antibodies:<br>- CD3, CD4, CD28: BD Biosciences website - Reactivity: Human (QC testing); staining is shown on human peripheral blood lymphocytes; routinely tested by flow cytometry<br>- CD8, CD95: BD Biosciences website - reactivity Human (QC testing), Rhesus, Cynomolgus, Baboon (Tested in Development); staining is shown on human peripheral blood lymphocytes; routinely tested by flow cytometry<br>- CD45RO: BD Biosciences website - Reactivity: Human (QC testing); staining is shown on human lysed whole blood; routinely tested by flow cytometry<br>- CD25: BD Biosciences website - reactivity Human (QC testing), Rhesus, Cynomolgus, Baboon (Tested in Development); staining is shown on PHA-stimulated human peripheral blood lymphocytes; routinely tested by flow cytometry<br>- CD103, CD278/ICOS, CD134/OX-40: BD Biosciences website - Reactivity: Human (QC testing); staining is shown on phytohemagglutinin-stimulated human peripheral blood mononuclear cells; routinely tested by flow cytometry<br>- CD154/CD40L: BD Biosciences website - Reactivity: Human (QC testing), Rhesus, Cynomolgus, Baboon (Tested in Development); staining is shown on PMA and Calcium Ionophore A23187 stimulated human peripheral blood mononuclear cells; routinely tested by flow cytometry<br>- CD152/CTLA4 BD Biosciences website - reactivity Human (QC testing), Rhesus, Cynomolgus, Baboon (Tested in Development); staining is shown on Concanavalin A-activated human peripheral blood mononuclear cells; routinely tested by flow cytometry<br>-CD274/PD-L1: BD Biosciences website - reactivity Human (QC testing); staining shown on MIT76 transfected cells; routinely tested by flow cytometry<br><br>-CCR7, CD127: BioLegend website - Reactivity: Human, African Green, Baboon, Cynomolgus, Rhesus; staining shown in human peripheral blood lymphocytes<br>- PD-1: BioLegend website - Reactivity: Human, African Green, Baboon, Chimpanzee, Common Marmoset, Cynomolgus, Rhesus, Squirrel Monkey; staining shown in human peripheral blood lymphocytes<br>-CD3: BioLegend website - Reactivity: Human, staining shown in human peripheral lymphocytes<br>-CD4: BioLegend website - Reactivity: Human and chimpanzee; staining shown in human peripheral blood lymphocytes<br>- 4-1BB: BioLegend website - Reactivity: Human and Cross-reactivity: Chimpanzee, Baboon, Cynomolgus, Rhesus; staining shown in PHA-stimulated human peripheral blood mononuclear cells.<br><br>-CD45RA: Beckman Coulter website - Target Species: Human; antibody was evaluated during the 3rd and the 4th HLDA workshop on Human Leukocyte Differentiation in Oxford (1986) and Vienna (1989) respectively. 2H4LDH11LDB9 (2H4) monoclonal antibody is restricted to the CD45RA antigen.<br><br>-CD27: LifeTechnologies website - Species Reactivity: Human; This O323 antibody has been pre-titrated and tested by flow cytometric analysis of normal human peripheral blood cells.<br><br>- LAG-3, TIM-3: R&D Systems website - Species Reactivity: Human; staining shown in human peripheral blood monocytes<br><br>In addition to above, the following samples were used as controls to ensure appropriate staining for each flow cytometry run containing clinical samples: unstimulated healthy donor peripheral blood mononuclear cells and NY-ESO-1 specific TCR transduced cells as a control for NY-ESO-1 Pentamer staining.<br><br>The following validation information is available for IHC antibodies.<br><br>- NY-ESO-1: Millipore website (acquired Sigma) - Reactivity: human; Custom validation performed on human testis, ovarian carcinoma,non-small cell lung cancer, sarcomas, melanoma, and normal tissue array<br>- CD4: Roche website (acquired Ventana) - Quality Control Tissues: Liver and tonsil for each antibody lot

- CD8: Dako/Agilent website - Specificity: SDS-PAGE analysis of immunoprecipitates formed between lysates of 125I-labeled human T lymphoblasts and the antibody shows reaction primarily with a 32 kDa polypeptide corresponding to CD8α (1). See package insert for reference(s).; IHC staining in human tonsil, spleen, and peripheral T-Cell lymphoma shown
- CD20: Roche website (acquired Ventana) - Quality Control Tissues: Lymph Node and Tonsil for each antibody lot
- CD45: Agilent webiste - Specificity: Anti-CD45 is a mixture of two monoclonal antibodies, clones 2B11 and PD7/26, directed against different epitopes. Clone 2B11 was clustered as anti-CD45 at the Third International Workshop and Conference on Human Leucocyte Differentiation Antigens, held in Oxford in 1986 and reacts with all the known isotypes of the CD45 family. Clone PD7/26 was clustered as anti-CD45RB at the Fifth International Workshop and Conference on Human Leucocyte Differentiation Antigens, held in Boston in 1993. See package insert for reference(s); IHC staining in acute myeloid leukemia, cerebellum, tonsil, and B-cell chronic lymphocytic leukemia/small lymphocytic lymphoma shown.
- CD163: Roche website (acquired Ventana) - Quality Control Tissue: Inflamed Tissue for each antibody lot
- LAG-3: Novus Biological website - Reactivity: Human, Primate reactivity reported in scientific literature (PMID: 32284611); IHC staining in human tonsil tissue shown
- PanCK: Roche website (acquired Ventana) - Quality Control Tissues: Intestine, Liver, and Skin for each antibody lot
- PD-1/CD279: Abcam website - Reactivity: Human; Positive Controls for IHC: Human Tonsil Tissue
- PD-L1: Roche website (acquired Ventana) - Quality Control Tissue: Tonsil for each antibody lot
- TIM-3: Cell Signaling Technologies website - Reactivity: Human; IHC staining in renal clear cell carcinoma shown

Human research participants

Policy information about studies involving human research participants

Population characteristics	Study included 45 patients with advanced or metastatic synovial sarcoma that were treated with lete-cel. Across the 4 cohorts, 21 patients were women and 24 patients were male. The median age was 32 years old. With regards to ECOG performance status, 25 patients had a score of 0 and 19 patients had a score of 1. All patients received prior systemic therapy and 15 patients received bridging therapy between leukapheresis and the start of lymphodepletion, followed by cell infusion. At minimum the duration between bridging chemotherapy and lymphodepletion was the protocol specified washout chemotherapy of at least three weeks for cytotoxic chemotherapy, immune therapy, and monoclonal antibody therapy, and at least one week or 5 half-lives for approved targeted small molecular inhibitors or biologics.
Recruitment	Patients were recruited at the participating clinical trial centers utilizing the site specific standard mechanisms for clinical trial recruitment. Selection was based on principal investigator assessment of inclusion and exclusion criteria. There were no other known selection biases present. It is important to note that synovial sarcoma is a rare population. All patients signed a screening consent form to evaluate HLA genotype and NY-ESO-1 expression. Once confirmed and all other inclusion and exclusion criteria were met, patients signed treatment consent form.<br><br>Written informed consent was obtained from each subject prior to the performance of any study-specific procedures. The investigator agreed to provide the subject sufficient time to review the document, to inquire about details of the trial, and to decide whether or not to participate in the study. The informed consent was signed and dated by the study subject and by the person who conducted the informed consent discussion. The informed consent for pediatric subjects was signed and dated by the parent or legal guardian of the study subject and by the person who conducted the informed consent discussion. No participant compensation was given except for travel/lodging expenses reimbursement.
Ethics oversight	The study was conducted in accordance with the Declaration of Helsinki and Good Clinical Practice guidelines following approval by ethics committees and institutional review boards at each study site (see below). All patients provided written informed consent.<br><br>- National Cancer Institute, Bethesda, MD: National Institutes of Health Intramural Institutional Review Board<br>- Memorial Sloan Kettering Cancer Center, New York, NY: Memorial Sloan Kettering Cancer Center Institutional Review Board/Privacy Board<br>- MD Anderson Cancer Center, Houston, TX: The University of Texas M.D. Anderson Cancer Center Institutional Review Board<br>- City of Hope Comprehensive Cancer Center, Duarte, CA: City Of Hope Institutional Review Board<br>- H. Lee Moffitt Cancer Center, Tampa, FL: University of South Florida, Research Integrity & Compliance Office Institutional Review Board-Human Research Protection Program<br>- Dana-Farber Cancer Institute, Boston, MA: Dana Farber Cancer Center Institutional Review Board<br>- Washington University in St. Louis School of Medicine, St. Louis, MO: Washington University School of Medicine in Saint Louis, Department Human Research Protection Office (HRPO)<br>- Division of Oncology, Children's Hospital of Philadelphia and University of Pennsylvania, Philadelphia, PA: Office of Human Subject Protection, The Children's Hospital of Philadelphia

Note that full information on the approval of the study protocol must also be provided in the manuscript.

Clinical data

Policy information about clinical studies

All manuscripts should comply with the ICMJE guidelines for publication of clinical research and a completed CONSORT checklist must be included with all submissions.

Clinical trial registration	NCT01343043
Study protocol	The study protocol can be found on Clinical Trials.gov: https://clinicaltrials.gov/ct2/show/NCT01343043/
Data collection	Patients were enrolled at 8 centers within the US (see below). The study period, including screening, enrollment, treatment and follow-up, was from September 27, 2012 to June 18, 2019.

- National Cancer Institute, Bethesda, MD
- Memorial Sloan Kettering Cancer Center, New York, NY
- MD Anderson Cancer Center, Houston, TX
- City of Hope Comprehensive Cancer Center, Duarte, CA
- H. Lee Moffitt Cancer Center, Tampa, FL
- Dana-Farber Cancer Institute, Boston, MA
- Washington University in St. Louis School of Medicine, St. Louis, MO
- Division of Oncology, Children's Hospital of Philadelphia and University of Pennsylvania, Philadelphia, PA

All research specimens were collected at the clinical sites above. Biomarker testing was performed at the following locations:

- Cell Expansion and Cytokine Analyses: Cambridge Biomedical (now part of BioAgilytix), Boston, MA
- Flow Cytometry: Caprion (now part of CellCarta), Montreal, Canada
- Tumor RNA gene expression and IHC analyses: Histogenex (now part of CellCarta), Wilrijk, Belgium
- Tumor NY-ESO-1 IHC analysis: QualTek Laboratory (now part of Discovery Life Sciences), Goleta, CA
- Tumor RNAScope analysis: Advanced Cell Diagnostics, Newark, CA

Outcomes

The primary efficacy outcome was investigator-assessed objective response rate (ORR; complete response or partial response) per Response Evaluation Criteria in Solid Tumors (RECIST) v1.1. Secondary efficacy outcomes included duration of response (DoR), progression-free survival (PFS), best overall response, and overall survival (OS).

Secondary safety endpoints were included adverse events (AEs), serious AEs (SAEs), and AEs of special interest (AESI); all were evaluated using Common Terminology Criteria for Adverse Events v4.0 (CTCAE v4.0).

Exploratory biomarker endpoints included correlation of expansion, phenotype, and functionality of lete-cel in the blood and or tumor with response to treatment as well as correlation of biomarkers in tumor tissue and blood with response following infusion of lete-cel.

Flow Cytometry

Plots

Confirm that:

☒ The axis labels state the marker and fluorochrome used (e.g. CD4-FITC).

☒ The axis scales are clearly visible. Include numbers along axes only for bottom left plot of group (a 'group' is an analysis of identical markers).

☒ All plots are contour plots with outliers or pseudocolor plots.

☒ A numerical value for number of cells or percentage (with statistics) is provided.

Methodology

Sample preparation

Peripheral blood mononuclear cells (PBMCs) were collected at the listed timepoints from each patient in CPT tubes, centrifuged, and sent to central lab. Central lab washes cells in PBS followed by cryopreservation. For analysis, PBMCs were thawed (1x106 cells/panel) and incubated with Fc blocker (for Pheno2 only) for 10 minutes at room temperature, prior to being washed and subjected to pentamer staining (10 minutes at room temperature). PBMCs were then washed and stained with surface stainer (30 minutes at 4°C), then washed again and fixed in 0.5% paraformaldehyde (30 minutes at 4°C). Cells were then washed again before acquisition.

Instrument

Data was acquired on LSR II (BD Biosciences)

Software

Raw data files (.fcs) were analyzed using FlowJo software v9.9(BD).

Cell population abundance

Cells were gated on single, live CD3+ cells (see below) and only samples with >5,000 viable, CD3+ cells were included in the analysis to ensure robust data. Most samples had >50,000 viable, CD3+ cells.

Gating strategy

Analyses were performed on live, singlet CD3+ T cells. Analyses on transduced T cells were performed on Pentamer+ cells. . When the frequency for the Pentamer+ parent gate for a sample was lower than the noise level determined from the maximum value for all apheresis samples (in which there should not be any Pentamer+ cells), we applied a flooring by setting the frequency of the parent gates to 0 and subsequent children gates to 'not available' for all samples. This flooring was separately done for each CD8+Pentamer and CD4+Pentamer+ populations. Examples of gating strategy for Pheno 1 and 2 are shown in Supplementary Figure S9.

☒ Tick this box to confirm that a figure exemplifying the gating strategy is provided in the Supplementary Information.
